# Advancements in Imaging and Neurosurgical Techniques for Brain Tumor Resection: A Comprehensive Review

**DOI:** 10.7759/cureus.72745

**Published:** 2024-10-31

**Authors:** Nidhi H Vadhavekar, Tara Sabzvari, Simone Laguardia, Thuslim Sheik, Varsha Prakash, Aseem Gupta, Indra Dhanush Umesh, Abhinandan Singla, Ikhlaq Koradia, Brando B Ramirez Patiño, Humza F Siddiqui

**Affiliations:** 1 Department of Internal Medicine, D.Y. Patil University School of Medicine, Navi Mumbai, IND; 2 Department of Medicine, McMaster University, Hamilton, CAN; 3 School of Medicine, University of Milan-Bicocca, Monza, ITA; 4 Department of Surgery, Gulf Medical University, Ajman, ARE; 5 Department of Medicine, Sri Ramachandra Institute of Higher Education and Research, Chennai, IND; 6 Department of Medicine, University of St Andrews, St Andrews, GBR; 7 Department of Neurosurgery, Adichunchanagiri Institute of Medical Sciences, Mandya, IND; 8 Department of Internal Medicine, Government Medical College Amritsar, Amritsar, IND; 9 Department of Internal Medicine, Rajiv Gandhi Medical College, Thane, IND; 10 Department of Surgery, Universidad Michoacana de San Nicolás de Hidalgo, Morelia, MEX; 11 Department of Internal Medicine, Jinnah Postgraduate Medical Centre, Karachi, PAK

**Keywords:** brain tumor management, brain tumor surgery, minimally invasive surgery, neurosurgery advances, neurosurgical techniques, surgical outcomes.

## Abstract

Brain tumor surgery has witnessed significant advancements over the past few decades, resulting in improved patient outcomes. Despite these advancements, brain tumors remain a formidable public health challenge due to their high morbidity and mortality rates. This review explores the evolution of neurosurgical techniques for brain tumor resection, emphasizing the balance between minimizing invasiveness and maximizing precision. Traditional approaches like craniotomy and keyhole surgery remain crucial, but the rise of minimally invasive techniques such as endoscopic endonasal surgery and laser interstitial thermal therapy (LITT) has revolutionized the field. Awake craniotomy has been a substantial stepping stone towards the preservation of neurological function among brain tumor patients. Additionally, the integration of brain mapping technologies including intraoperative MRI, ultrasound and fluorescence-guided surgery has enhanced the precision of tumor resections, particularly in eloquent brain areas. These innovations, while promising, also come with challenges, including steep learning curves and limited access to advanced technology in certain regions. As the field progresses, ongoing research is essential to refine these techniques and improve accessibility, ultimately aiming to increase survival rates and preserve neurological function in patients with brain tumors. The integration of advanced imaging techniques refined surgical tools, and artificial intelligence (AI) in surgical planning is expected to further improve the safety and effectiveness of neurosurgical procedures in the future. This review provides a comprehensive analysis of current surgical strategies and explores potential future directions in brain tumor surgery.

## Introduction and background

Brain metastases are the most common type of intracranial tumor, although primary brain tumors are amongst the most fatal cancers which cause substantial morbidity and mortality [1]. Glioblastomas are considered as one of the most lethal cancers in the adult population [2]. The global incidence of primary brain tumors is approximately 10.82 per 100,000 person-years, with variations depending on the region and population studied [3]. The prevalence of brain tumors has been increasing, partly due to advances in diagnostic imaging techniques and increasing age, leading to a growing burden on the healthcare system [4]. Despite the overall rarity, the mortality rate for malignant brain tumors remains high, with five-year survival rates for glioblastomas, one of the most aggressive types, being around 6.8% [5]. High-grade gliomas have been found to have a median overall survival (OS) of 14 to 20 months, even after optimal multimodal therapy [6]. Medulloblastoma on the other hand is one of the most common pediatric brain tumors [2]. Primary neoplasms including lung cancer, breast cancer, and melanoma are most likely to metastasize to the brain [4, 5].

Neurosurgery plays a crucial role in the removal of brain tumors and the techniques have evolved significantly in the last few decades leading to improved patient outcomes in terms of both neurological function and survival rates. Traditionally it was open craniotomy procedures which were the standard approach for tumor removal, but they carried a higher risk of complications along with extensive brain tissue disruption. However, the newer minimally invasive techniques aim to reduce the invasiveness of the procedure while achieving optimal tumor resection. Minimally invasive techniques involve the use of smaller incisions, and specialized instruments, along with advanced imaging techniques to access and remove brain tumors. They aim to minimize the damage to healthy brain tissue surrounding the tumor, thereby preserving neurological function and reducing the risk of postoperative deficits along with reduced postoperative pain, shorter hospital stays, faster recovery, and improved cosmetic outcomes [7, 8].

Image-guided surgery utilizes preoperative imaging to create maps of the brain and guide the surgery. Therefore, allowing surgeons to visualize the tumor's location with respect to critical structures, thereby improving the safety and precision during resection. Intraoperative imaging techniques including intraoperative magnetic resonance imaging (iMRI) and real-time ultrasound, enable surgeons to assess the extent of tumor removal and make any further intraoperative adjustments enhancing the likelihood of achieving a complete resection. Fluorescence-guided surgery, often using 5-aminolevulinic acid (5-ALA), is another technique used for improved tumor visualization. Nuclear methods including positron emission tomography probes provide tumor detection based on beta or gamma emission after a radiotracer injection. Whereas spectroscopy-based methods offer molecular insights [7-10].

**Figure 1:**
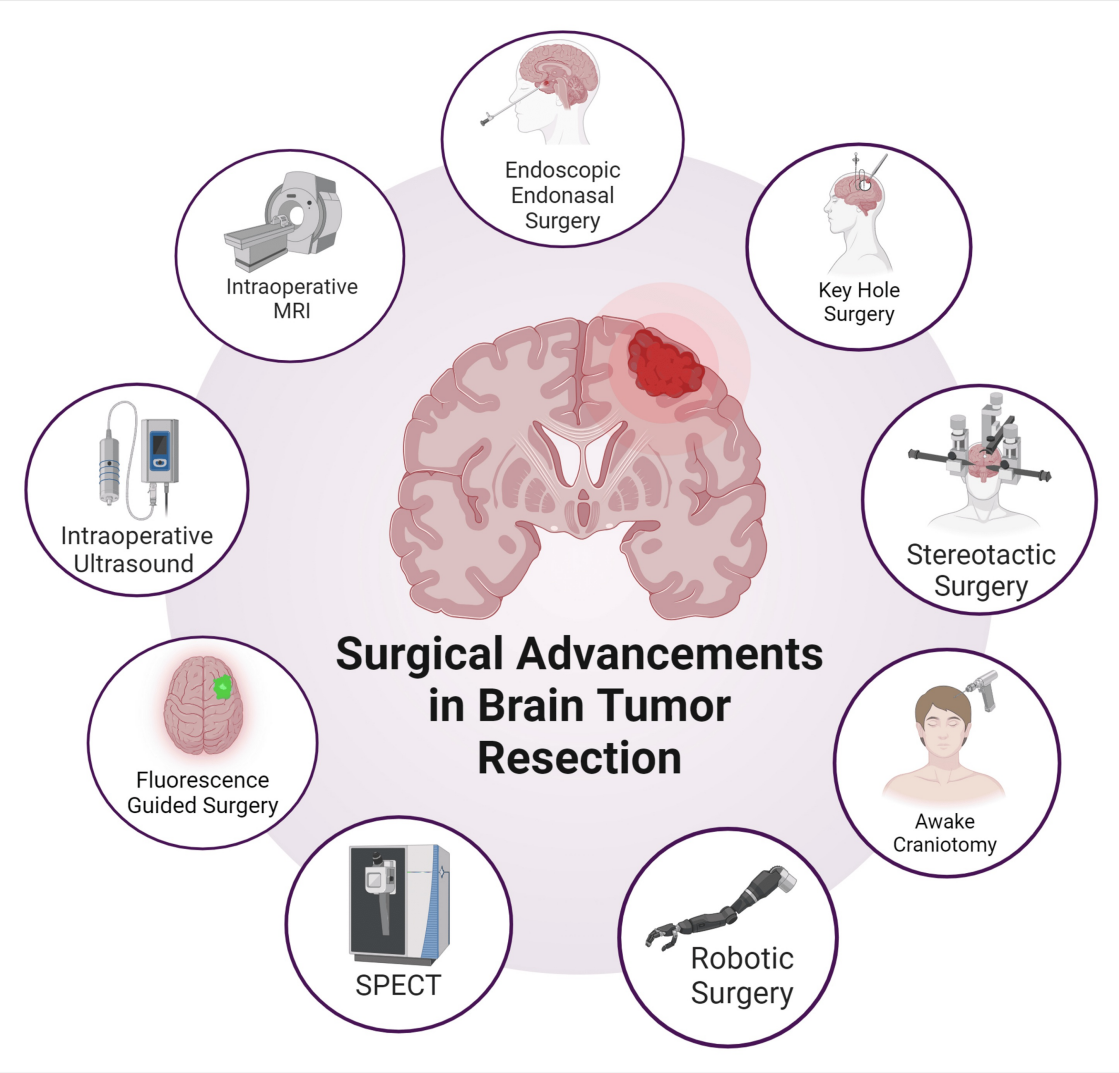
Overview of radiological and surgical advancements in neurosurgery for brain tumor resection Figure has been made using biorender.com

Among the foundational techniques, craniotomy and traditional keyhole surgery remain cornerstone procedures. Craniotomy, the surgical removal of part of the skull to access the brain, provides a wide exposure and is often used for larger or more complex tumors [11]. In contrast, the keyhole approach, which utilizes smaller incisions, aims to reduce tissue disruption and improve recovery times while still providing sufficient access for tumor resection [12]. The advent of endoscopic endonasal and intraventricular surgeries has revolutionized the management of tumors located near or within the ventricles and the base of the skull [13, 14]. Awake brain surgery has introduced a paradigm shift in the treatment of tumors located in eloquent areas of the brain. By performing the surgery while the patient is awake, surgeons can monitor and preserve critical neurological functions such as speech and motor skills in real-time, thereby reducing the risk of permanent deficits [15]. Stereotactic radiosurgery (SRS) delivers highly focused radiation to tumors with sub-millimeter precision without impacting the surrounding tissue and has emerged as a powerful non-invasive alternative for patients with small, inoperable, or recurrent tumors [16]. Recent innovations such as laser interstitial thermal therapy (LITT) and the tubular retractor system (TRS) have further expanded the arsenal of neurosurgeons [17, 18]. An overview is given in Figure 1.

This article aims to provide a comprehensive analysis of the advancements in these neurosurgical techniques, examining their impact on neurological function and survival rates, and exploring the potential future directions in the field.

## Review

Imaging and brain mapping modalities

Intraoperative Magnetic Resonance Imaging (iMRI)

The most common primary cancerous tumor is glioblastoma (GBM) with a high rate of recurrence and having an average of two years of survival rate. The surgically accessible tumors when resected aggressively with the guidance of iMRI have significantly helped in removing tumors by providing real-time visualization of the constant changes in the brain tissue that occur during the surgical procedure and it also helps to slow down the tissue infiltration rate. However, the accessibility of real-time intraoperative brain images and problems like shifting of the brain couldn’t be known by conventional neuronavigational techniques (CNN) which severely affected the patient’s outcome. Therefore, several techniques which help in minimizing the damage to brain tissue along with improving the extent of resection have come into practice [19].

Surgical results have significantly improved by taking the aid of iMRI which gives the advantage of real-time imaging leading to improvement in the extent of resection. iMRI eliminates the risk of major complications and additionally provides precise surgical interventions which makes it a safe tool for patients [20]. Whenever intraoperative evaluation is required for resection of brain tumor, iMRI remains the first choice of surgeons. Since the availability of iMRI machines is limited, a commercially available software, i.e. virtual MRI cranial, seems to be of good diagnostic accuracy in low-grade gliomas; by fusion of iMRI and iCT it predicts the left-over remains of a tumor with high sensitivity [21].

The extent of glioma resection can be maximized without causing any major obstruction in the surgical and anesthesiologic procedure and can be done in a short amount of time with the help of intraoperative low-field MRI [22]. With experienced neurosurgeons and anesthesiologists, the tumor resection can be maximized along with preservation of neurological functions by combining iMRI with awake craniotomy along with language mapping. It will be beneficial for the patients especially with tumors close to speech areas [23]. Advance technologies like 3-Tesla MRI helps the neurosurgeon to resect any remaining tumor that is safely accessible by improving the diagnostic quality scans and also alerts about brain parenchyma shifts [24].

Intraoperative Computed Tomography (iCT) Scan

The task to achieve good results along with decreased complications and other medical care are some of the challenges faced by neurosurgeons as modern neurosurgery demands the best treatment and disease control along with functional integrity to improve patient outcomes. In the multitude of intraoperative imaging methods, iCT is highly valuable [25]. In case of gliomas, there are high chances of inaccuracy while differentiating the tumor from surrounding tissue, it can be resolved by acquiring and displaying timely information during the resection of a glioma tumor and it can also provide access about the brain shift of less than an inch which can occur after craniotomy leading to several complications.

As there is a high risk associated with CT scans due to high radiation exposure as well as it is not affordable by many, an advanced version called multidetector CT or cone-beam CT provides better bony resolution with low radiation exposure as well as it is cost-effective [26]. The accuracy of navigation and brain shift management can be improved when iCT is combined with advanced image fusion algorithms [26].

To improve the intraoperative awareness and simplify the procedure of laser position, mobile CT scanner was used with laser interstitial thermal therapy (LITT) for removal of brain tumors, along with streamlining the procedure. It also helps to improve patients’ safety and comfort which increases patient compliance [27]. As compared to standard registration CT, iCT reduces the risk of radiation exposure when used for frameless biopsies. When the lesion is located near sensitive or dangerous areas of the brain or when small target volumes are required for biopsies, use of iCT is mostly preferred [28, 29].

Intraoperative Ultrasound (IOUS)

In the last 10 years, intraoperative ultrasound (IOUS) has developed into a widely used tool for neuroimaging that guides during surgery in real-time. It is an easily accessible imaging method with low cost, possesses minimal risk, and requires minimal additional operative time. In terms of identifying brain tumors and their anatomical relationships, it offers high-quality real-time images that facilitate control of the surgical process [30, 31].

The primary advantage of IOUS in brain surgery is its capability to provide imaging in real-time. It can quickly (within seconds) offer a comprehensive view of the tumor and its surrounding structures. Intraoperative ultrasound (IOUS) facilitates the repeated evaluation of tumor extent and location during surgery without extending the duration of the procedure [32]. The real-time anatomical data it provides is crucial for surgical decision-making. Intraoperative B-mode ultrasound enables more frequent and thorough resections compared to traditional methods, without additional risk to patients, making it a valuable tool for optimizing glioblastoma resection [33, 34]. In the study performed by Elmesallamy, IOUS demonstrated superior delineation of tumor characteristics compared to both CT and MRI T1. It significantly improved the definition of brain tumors regarding tumor edges, necrosis, contours, and cystic components. IOUS was as good as CT at identifying calcification, hematomas within tumors, and brain edema. Additionally, IOUS outperformed MRI T1 in detecting intra-tumoral calcification and was similarly effective as MRI T1 in identifying edema and hematoma [35].

Furthermore, advanced ultrasound techniques such as Doppler, contrast-enhanced ultrasound (CEUS), and elastography offer the potential to more precisely identify residual tumor volumes during surgical resection [36, 37]. Color Doppler (CD) and Power Doppler (PD) are two main types of Doppler used in oncological neurosurgery. Color Doppler (CD) provides a visual map of blood flow superimposed on B-mode imaging, allowing for better visualization during surgery. Three-dimensional (3D) iUS color Doppler detects brain shifts during surgery, thus improving patient safety. However, it is angle-dependent and unable to detect flow perpendicular to the ultrasound beam direction. Integrating the reconstructed vessel tree into the navigational system could enhance navigational updates and surgical accuracy. 3D Color Doppler cannot resolve small peritumoral vessels as effectively as pre-MRI due to its angle dependency. Power Doppler is a better alternative as it measures signal amplitude, not velocity, making it more effective for detecting small vessels and less affected by artifacts like angle dependency or aliasing. Ultrasensitive US Doppler techniques assess the perfusion pattern and capillary angioarchitecture of the tumor and the surrounding tissue [37-41].

CEUS offers real-time information on microvascular blood flow, enhancing tumor detection and aiding in distinguishing between benign and malignant tumors [26]. CEUS is characterized by rapid contrast enhancement with a fast arterial phase and a peak in contrast enhancement, followed by intense and sustained contrast enhancement with a delayed venous phase and an irregular, heterogeneous contrast enhancement pattern [40]. It can help distinguish benign from malignant gliomas during surgery. Also, it improves the visualization and boundary definition of lesions through vascular perfusion compared to B-mode ultrasound, thus significantly enhancing the identification of residual tumors and improving the total resection rate [42, 43].

Superb microvascular imaging (SMI) uses an adaptive algorithm to study motion artifact characteristics and remove clutter signals for low-speed microvascular visualization at higher frame rates and with more excellent spatial resolution without the need for contrast media enhancement. SMI provides high-quality boundary imaging, particularly for high-grade glioma (HGG) boundary identification in highly aggressive tumors, which is crucial for guiding surgical plans and adjusting treatment strategies. It enhances the presentation of brain tumors and provides histological information about vascularization, aiding neurosurgeons in distinguishing edema from tumors. This confirms the advantages of SMI over B-mode ultrasound in boundary delineation. Identification of residual tumors through intraoperative ultrasound detection and pathological biopsy are critical areas for further research. Furthermore, a new liquid acoustic coupler could emerge as a novel method to enhance the identification of residual tumors through ultrasound [44, 45].

Elastosonography tells us about the mechanical properties of tumors compared to the surrounding normal brain tissue. Algorithms based on elastosonography are more precise than B-mode ultrasound to differentiate between high-grade gliomas (HGGs) and metastases. Two main methodologies are used: strain elastography (SE), which applies a mechanical force to assess lesion stiffness and provide a color scale for qualitative analysis, and shear wave elastography (SWE), which offers a quantitative assessment by using an ultrasound stimulus to produce tissue movement, thereby offering a quantitative measure of stiffness. Elastosonography is particularly useful for characterizing gliomas and meningiomas [46-48].

Despite the great potential of multiparametric ultrasound (mpUS), its application is hindered by several limitations. One significant challenge is the steep learning curve, often more demanding than that of standard B-mode ultrasound, highlighting the need for targeted courses and resources. Furthermore, research in this area is still in its early stages and has yet to fully demonstrate the extensive benefits and applications of mpUS. To overcome these obstacles, new approaches are being explored, such as the use of specialized coupling fluids, micro-probes for internal cavity examination, contrast-enhanced ultrasound (CEUS), navigated ultrasound, and utilizing surrounding healthy brain tissue to enhance the study of the surgical cavity [40, 49].

Functional Magnetic Resonance Imaging (fMRI)

Tumor resection has been made easier with various imaging modalities like fMRI as the brain anatomy varies depending on different types of tumors and their compression. One of its main uses is in pre-surgical planning for brain tumor patients. By mapping critical brain functions such as motor skills, language and sensory processing, fMRI helps neurosurgeons plan resections to maximize tumor removal while minimizing damage to important brain areas. This functional mapping is crucial in cases where tumors are near eloquent brain regions, thus guiding the surgical approach and reducing postoperative deficits. There are two types of fMRIs: task-based fMRI (t-fMRI) and resting-state fMRI (rs-fMRI) [50].

Task-based fMRI measures brain activity while a person performs a task, then compares it to a predicted model of how the brain should function during that task. By using statistical methods, they can determine which areas of the brain are active and responsive. The experiment usually involves presenting sensory cues such as visual or auditory stimuli to guide the participant’s behavior while brain scans are taken. Capturing these brain responses within a single scan ensures the results are accurate and not affected by unrelated changes in the participant or the scanning equipment [51].

There are three types of task-based fMRI designs done. The first is the block design, where the patient is made to perform tasks repeatedly in an alternating manner. It is mainly used to identify the areas involved in a certain task but fails to differentiate the responses for each task. Secondly, the event-related design is performed, wherein tasks are presented individually and randomly and enough time is given between tasks to analyze and study the brain’s responses to them. This is highly preferred for studying cognitive abilities and actions; however, it produces a very soft signal quality. Lastly, the mixed design is performed, which combines both block and event-related tasks. This shows short-term and long-term brain activity [52].

Resting-state fMRI is a brain imaging technique that looks at natural brain activity when a person is at rest and not doing any specific task. It measures low-frequency signals in the brain which reflects the spontaneous neural activity happening in different brain regions. This method helps researchers understand how different parts of the brain communicate with each other during rest. Unlike task-based fMRI, it doesn’t require the individual to do anything specific during the scan [53].

Some studies show the advantages of using resting-state fMRI (rs-fMRI) over task-based fMRI (t-fMRI), as rs-fMRI can assess multiple brain functions in one session without requiring active patient involvement, and hence is being used as a standard tool for neurological planning especially for patients that cannot cooperate in t-fMRI. While task-based fMRI provides valuable preoperative mapping for brain tumor resection, it could have limitations in patients who cannot cooperate, like children or patients with neurocognitive disabilities [54]. Rs-fMRI was shown to reveal alterations in brain connectivity during resting state in cases of gliomas, glioblastomas, and pituitary adenomas, which then disrupted functional connectivity causing cognitive impairment in these patients [55]. It was innovated with another novel imaging analysis method named Regional Parameter of Resting-state fMRI-omics (RP-Rs-fMRIomics), which significantly improved the prediction of glioma grade, IDH genotype, and prognosis compared to traditional rs-fMRI approaches [56]. With regard to patient outcomes and pre-planning before tumor resection, rs-fMRI proved more effective than task-based fMRI in identifying the ventral somatomotor network in brain tumor patients, which is very crucial for fine motor control and coordination of movements, especially in the lower face and upper body regions. However, task-based fMRI cannot be completely replaced by rs-fMRI [57].

Task-based fMRI is also a very valuable tool for surgical planning; however, its reliability depends upon the grading of the tumor. In tumors like glioma, this type of fMRI is sensitive in the detection of both high-grade and low-grade gliomas, however, its specificity is lower in high-grade glioma patients, and reliable in cases of low-grade gliomas [58]. Task-based fMRI has also been shown to aid in pre-operative surgical planning as it localizes critical functional areas like the somatosensory, motor, and language regions helping to balance tumor resection with minimal functional loss. Although it has the potential to replace invasive diagnostic tools, its integration with other modalities like Diffusor-Tensor Imaging and Tractography (DTI & DTT) is vital and improves the specificity of the surgery [59].

A study emphasizes the effectiveness of task-based fMRI in accurately identifying motor and language regions before surgery for individuals with brain tumors, whereas Rs-fMRI offers valuable information about the brain’s functional structure without requiring patient cooperation [51]. Conversely, another study highlights that t-fMRI shows brain activity during tasks while rs-fMRI looks at spontaneous brain activity making it helpful for patients who can’t complete tasks. However, the reliability of rs-fMRI is influenced by changes caused by tumors and challenges in data processing, hence more research is required to stick with a single approach. However, when used in conjunction with other imaging modalities like MRI tractography and direct cortical stimulation (DCS) during surgery, it can improve planning and potential outcomes. However, evidence regarding its impact on survival and functional results is still limited [60]. Despite all these studies, a majority of the literature available is looking forward to accentuate the usage of rs-fMRI as an additional tool in the preoperative assessment process aiming to enhance the efficiency and accuracy of tumor removal procedures [61].

Positron Emission Tomography (PET)

Positron emission tomography has become a very valuable and essential tool in the field of neuro-oncology for various reasons. Especially, PET using amino acid-based tracers has visualized tumors more effectively than other conventional methods, with increased accuracy, which is a great milestone in this field. PET with specific tracers improves the ability to detect tumors even in the hardest-to-reach parts of the brain, like the base of the skull, and is able to actively differentiate between scar tissue and active tumor tissue [62].

A study published suggested that 18F-FACBC PET/MRI could be an important tool for distinguishing between low- and high-grade gliomas. This study found that 18F-FACBC PET/MRI significantly improves the detection of gliomas compared to MRI alone. PET/MRI had a 100% detection rate for lesions, and PET tumor volumes were generally larger and more accurate in identifying malignant tissue than MRI. This study concludes by mentioning that 18F-FACBC PET is well-suited for guiding tissue sampling and tumor resection because it accurately identifies areas with high tumor grade, cell proliferation, and cell density in PET-positive regions [63].

Another study emphasized PET, specifically 68Ga-DOTA-TATE PET combined with computed tomography (CT), and stated that this combination has shown better performance in visualizing and identifying leftover tumor tissue in various neuromalignancies like gliomas, meningiomas, and glioblastomas compared to CT-MRI, hence helping in complete tumor resection [64]. PET imaging plays a role in identifying and removing brain tumors especially when assessing tumor activity in areas where the blood-brain barrier (BBB) is intact or only partially intact. This makes CT and MR imaging methods less effective. The use of 18F fluorodeoxyglucose (FDG) PET has been established for purposes such as detecting tumors, defining their boundaries, monitoring recurrences and predicting prognosis with increased FDG uptake associated with tumor grades. Although FDG is the tracer used for these procedures, other PET tracers are currently being developed to enhance brain tumor evaluation although more research is required, before they can be widely used in clinical settings. Other PET tracers that target somatostatin receptors (SSTRs) play a role in neurotransmission and inhibiting cell growth. Somatostatin interacts with five subtypes of SSTRs and PET imaging using 68Ga-DOTA peptides (68Ga-DOTA-TOC, 68Ga-DOTA-NOC, and 68Ga-DOTA-TATE) which have a strong affinity for SSTR2 enables the assessment of SSTR expression in different tumors such as gliomas, meningiomas, primitive neuroendocrine tumors and medulloblastomas, and they show varying levels of SSTR expression making this imaging technique valuable for evaluating tumors [65].

Even a combination of modalities like MRI with PET can prove to be valuable in tumor detection. The use of multiparametric PET/MRI combined with the amino acid analogue O-(2-18F-fluoroethyl)-L-tyrosine-(18F-FET) allows for a comprehensive evaluation of brain tumors based on their molecular, structural and functional features. A study reported that multiparametric 18F-FET PET/MRI showed effectiveness in detecting and removing brain tumors, and achieved an accuracy rate of 85% for identifying cancer at the initial diagnosis reflecting high specificity, and 93% in detecting genuine progression during treatment indicating a very impressing level of sensitivity. These results highlight the dependability of 18F-FET PET/MRI in differentiating between tumor advancement and imaging alterations related to therapy which can greatly influence clinical decision making [66].

Assessing changes in metabolic tumor volume (MTV) through amino acid PET scans has also emerged as a valuable method for evaluating treatment responses in individuals with brain tumors. Automated segmentation of metabolic tumor volume (MTV) using 18F-FET PET demonstrates high sensitivity and specificity in brain tumor detection and response assessment. This technique successfully recognized 92% of tumors with heightened activity and 85% of those exhibiting normal or reduced activity levels aligning closely with manual segmentation performed by seasoned doctors. Moreover, the alterations in MTV detected through this method were significant indicators of both disease-free survival and overall survival rates in glioma patients [67].

Tumor-associated neuroinflammation can also be studied using a combination of PET tracers and MRI, which is enhanced with ultrasmall superparamagnetic iron oxide (USPIO) nanoparticles and can assist in spotting neuroinflammatory elements. Whereas PET holds the potential for assessing tumor antigen expression, drug delivery and therapeutic efficacy. Upcoming studies seek to merge new PET radiotracers and MRI contrast agents with approaches to boost the accuracy of neuroinflammation imaging and refine personalized treatment approaches [68]. Another PET tracer namely, [18F]BBPA, a boronophenylalanine-based PET tracer, in brain tumor imaging shows significant accumulation in tumors with a high tumor-to-normal brain ratio, positioning it as a promising candidate for PET imaging and boron neutron capture therapy (BNCT). MRI contrast enhancement does not influence its uptake, indicating its potential to enhance tumor-specific diagnosis and treatment [69].

This modality has several advantages and limitations, ranging from high sensitivity, and easy conjunction with other modalities to poor image resolution, and exposure to intensive radiation. A study underscores the drawbacks of using Fluorodeoxyglucose PET (FDG PET) for brain tumor imaging, mainly because of the interference caused by normal brain activity and difficulties in defining tumor edges. The findings indicate that combining FDG PET with MRI enhances diagnostic precision sharply when compared to combining FDG PET with CT. The use of amino acid PET especially Fluoroethyltyrosine (FET PET) is favored due to its superiority in distinguishing tumors from non-cancerous conditions and normal tissue and offering comprehensive information, about tumor size and advancement. It is suggested to use FET PET/MRI for its effectiveness in identifying tumor recurrence and for its ability to differentiate it from changes induced by treatment [70]. Even 11C-MET PET, another amino acid tracer, showed a high specificity rate in identifying tumor tissue in the brain, as it could distinguish pretreatment brain tumors from non-neoplastic lesions, hence a positive result could warrant further invasive testing like a biopsy [71].

Magnetic Resonance Spectroscopy (MRS)

Magnetic resonance spectroscopy (MRS) is a non-invasive technique that measures the concentration of specific biochemicals within the brain, providing insight into tumor metabolism. MRS is particularly useful intraoperatively for distinguishing between different types of brain tumors and identifying areas of active tumor growth and residual tumors [72].

Another study verified its usage preoperatively as well as intraoperatively. Preoperatively, MRS is able to measure the ratios of choline to N-acetylaspartate and choline to creatine. A lower-than-usual ratio suggests high-grade gliomas; hence, this technique effectively differentiates between different grades of the tumor. In intraoperative usage, MRS was effective at finding leftover tumor in 10 out of 12 cases where regular MRI suggested there might be some left. For high-grade tumors, MRS was able to clearly identify both types of remaining tumor, whether they showed up with contrast or not [73]. Hence, combining PET and MRS helps surgeons use special navigation systems to target tumor areas based on their activity and characteristics. This approach, shown with MRS and 18F PET, could also work with other imaging methods and tracers and can help identify areas with high tumor activity [74].

Newly emerging literature suggests that ultra-high field 7T MRS is highly effective in detecting various brain tumors and distinguishing them from low-grade to high-grade. It has been shown to be more sensitive and specific in detecting metabolites like D-2-hydroxyglutarate, glutamate, glutamine and glycine compared to MRS with lower field strength. While it holds promise for enhancing tumor assessment and tracking treatment progress its clinical adoption will take time [75]. Hydrogen proton magnetic resonance spectroscopy (1H-MRS) is another technique that has been successful in diagnosing and differentiating intracranial tumors, including meningiomas, gliomas, and metastases. 1H-MRS is able to provide insights into the biochemical composition of tissues without requiring any invasive procedures. It can differentiate between gliomas (LGG) and high-grade gliomas (HGG) by analyzing ratios of metabolites such as Cho/Cr and Cho/NAA. Additionally, it can assist in distinguishing HGG from metastatic tumors which can improve diagnosis and treatment planning [76].

MRS has also shown effectiveness in identifying the malignant transformation of low-grade gliomas, especially when there is heightened contrast enhancement. The study revealed that the NAA/Cho ratio serves as an indicator for malignant transformation exhibiting a sensitivity of 94.4% and a specificity of 83.3%. This technique has proven to be valuable alongside traditional imaging approaches to enhance the evaluation of tumor advancement [77]. The use of MR spectroscopy (MRS) along with other modalities like diffusion-weighted imaging (DWI) proves to be beneficial in assessing the growth of cells and the severity of gliomas before surgery, greatly improving the accuracy of diagnoses. Previous research emphasizes that MRS and DWI can effectively predict cell proliferation and glioma grade preoperatively. Combining MRS with DWI enhances sensitivity and specificity in grading gliomas, with MRS + DWI showing excellent diagnostic performance. DWI compensates for MRS limitations, and the use of Fisher's linear discriminant functions provides an effective model for distinguishing low-grade from high-grade gliomas, significantly increasing the quality of patient outcomes [78]. With regards to accuracy, MRS shows greater reliability than PET in distinguishing between tumor recurrence and radiation necrosis (RN) following whole brain radiation therapy (WBRT) or stereotactic radiosurgery (SRS). With a sensitivity of 100% and an accuracy rate of 81.8% MRS surpasses PET CT which has lower sensitivity and accuracy. The negative predictive value of MRS is especially useful, in differentiating recurrent tumors from radiation-induced changes [79].

To increase the accuracy of diagnosis of any brain tumor it is recommended to combine different modalities. The use of MRS alongside traditional MRI improves the accuracy of diagnosing brain tumors and classifying them according to their tumor grade. The analysis of Choline/Creatine and Choline/NAA ratios from MRS in conjunction with MRI resulted in a sensitivity of 87.5% and a specificity of 88.6% which is highly significant. This combined approach offers a diagnostic advantage enhancing the ability to distinguish between different types of brain tumors compared to relying solely on MRI’s visual field [80].

Intraoperative Fluoroscopy

In case of diffuse gliomas, even MRI and white light microscopy couldn’t distinguish the margins of the tumor from normal brain parenchyma, as the tumors infiltrate rapidly. To overcome this intraoperative fluoroscopy has been developed to accurately outline the tumor and maximize the extent of resection of high-grade gliomas (HGG). The use of 5-Amino levulinic acid (5-ALA) has been rapidly increasing since it has been approved by the American Food and Drug Administration (FDA). Compared to white light-based microsurgical resection, 5-ALA decreases the rate of neurological deficits along with increasing the rate of total gross resection, it is also reliable and cost-effective which makes it a strong independent predictor of survival [81]. Brain tumors can be caused to light up by use of intravenous fluorescein or oral 5-ALA which can be viewed through surgical optics by using appropriate filters and light of specific wavelengths [82]. Prediction of 5-ALA fluorescence helps in selecting the patient with diffuse gliomas. Enhancing and non-enhancing gliomas both have been reported to predict 5-ALA fluorescence. Fluorescence prediction has shown high efficiency in determining the spread of glioma when predicted by arterial spin labelling perfusion [83].

Since infiltrating and necrotic HGG both have different appearances still it is difficult to delineate the infiltrating regions of HGG from normal parenchyma as the mass of tumor decreases. When a tumor is situated near a sensitive area, selection of area of access becomes crucial. In order to protect the healthy cortex and find a specific site for resections, fluorescence is mostly used. The most common application of 5-ALA is to visualize the undetectable areas tumor infiltration in the brain. Therefore, some surgeons use fluorescent conditions for the whole procedure while some surgeons prefer using white light which helps in removing the tumor in one go and then use fluorescence to visualize any undetectable remains of the tumor. The most aggressive portion of the tumor lights up the most therefore selecting the fluorescing tissue helps to ensure the removal of the most violent portion of the tumor [84].

In fluorescence-guided surgery, fluorescein sodium is ideally used as it has 100% selectivity. With the use of white light illumination and standard neurosurgical microscope, sodium fluorescein can stain the tissue sufficiently for resection although there are some limitations regarding colour discrimination. Being used as a fluorescence marker and the simplicity, safety and easy availability for resection of intra-axial brain tumors, fluorescein sodium has been helpful in achieving total gross resection of brain tumors [85]. To perform an accurate targeted surgery and to find the cleavage plane around brain metastasis (BM), sodium fluorescein (SF) has been found to be helpful as it guides in localizing of subcortical BM. In case of biopsy for gliomas, SF is a good marker due to its ability of passing through the blood-brain barrier when it is compromised by a cancerous tutor, and it is cheaper also compared to other neuronavigational tools [86].

Single Photon Emission Computed Tomography (SPECT)

SPECT, similar to PET, is a form of nuclear imaging that enables the assessment of tissue perfusion and metabolic activity. It works by administering a radioactive tracer that binds to specific tissues through a tissue-targeted ligand. This radioactive isotope emits gamma rays, which are detected by a gamma camera [87]. SPECT, similar to other imaging modalities, can effectively differentiate between low-grade and high-grade gliomas. In a study of 61 patients with glioma, the technetium-99m sestamibi (99mTc-MIBI) SPECT index was significantly greater in high-grade gliomas compared to low-grade gliomas. Similar findings were observed with the 99mTc-glucoheptonate (GHA) radiotracer, which successfully distinguished low-grade gliomas, high-grade gliomas, and nonneoplastic lesions [88]. A comparative study by Kumar et al. demonstrated that [99mTc] Tc-methionine SPECT-CT is as effective as [11C] methionine PET in distinguishing gliomas from necrosis, particularly in high-grade gliomas [89]. Furthermore, SPECT has proven to be a valuable follow-up tool, with 99mTc-GHA SPECT and nitrogen-13 ammonia (13N-NH3) PET exhibiting high accuracy of 85.5% and 83.6%, respectively, in detecting recurrent gliomas [90].

Future research could focus on enhancing the precision and quantification of SPECT imaging by improving corrections for scattered radiation, attenuation, and spatial resolution loss. Optimizing reconstruction techniques to better account for count statistics and system calibration data may also yield improvements in spatial resolution and the quantification of clinically relevant parameters in early disease stages [91]. A study successfully demonstrated the potential of the INSERT (INtegrated SPECT/MRI for Enhanced Stratification of Brain Tumors in Radio-ChemoTherapy) system alongside MRI sequences required for SPECT operation. The imaging protocol successfully addressed challenges like field inhomogeneity and electrical interference, allowing for simultaneous acquisition, albeit with a restricted selection of MRI sequences. These early results suggest significant future advancements in SPECT-MRI integration [92].

Endoscopy

The endoscope has transformed into a precious visualization instrument in neurosurgery over the last three decades, being utilized in various neurosurgical procedures [93]. Incorporating the endoscope in an extended transnasal approach to brain tumors has transformed skull-base surgery [94]. Currently, endoscopy is used in multiple neurosurgical procedures, including third ventriculostomy, resection of intraventricular tumors, and transnasal approaches for pituitary and other skull-base tumors [93].

These endoscopic methods help neurosurgeons enhance tumor removal as they can expand the area of surgical dissection without enlarging the surgical approach size, consequently reducing perioperative morbidity due to surgical manipulation of critical brain structures. Endoscopy provides us with direct illumination of the surgical field, increased magnification, and the capability to visualize difficult corners using angled optics [93, 95]. A study done by El Beltagy and Atteya in 2020 demonstrated that endoscopy-assisted microsurgery for brain tumors was beneficial under challenging regions of the brain such as tumors of the cerebellopontine angle, ventricles of the brain, and craniopharyngioma, as it permitted closer examination of tumor extensions and surrounding vital tissues [96].

However, the major drawbacks of the endoscope include the lack of stereopsis, meaning it cannot provide true 3D vision, and motion parallax, which causes the illusion that objects closer to the viewer move faster than those farther away. Additionally, fish-eye image distortion can result in some degree of inaccurate 3D perception. The study by Uvelius and Siesjö, which compared 2D and 3D endoscopy, showed similar results based on basic outcome parameters [97]. However, some neurovascular injuries have also been documented during endoscope-assisted procedures [98]. A study by Sankhla et al. demonstrated endoport-guided endoscopic excision of interaxial brain tumors. The research showed that the technique is safe and provides an effective alternative for the removal of intraparenchymal and intraventricular tumors [99, 100].

Although already transformative, endoscopic neurosurgery continues to progress with emerging trends poised to redefine the future of the field. The integration of robotics is gaining popularity, offering improved precision and stability in delicate procedures. Augmented reality (AR) is being explored, and research is being made to provide surgeons with real-time, 3D views of the surgical field, potentially increasing spatial awareness [95, 100]. Table 1 summarizes the advantages and disadvantages of all imaging modalities.

**Table 1:** Summary of advantages and disadvantages of imaging modalities

Imaging Modality	Advantages	Disadvantages
Intra-operative magnetic resonance imaging (iMRI) [19-24]	Significantly enhances the extent of tumor resection. Provide real-time visualization of dynamic changes that occur during surgery. Provides high accuracy in low-grade gliomas. Helps in performing awake craniotomy with language mapping. Can predict the presence and absence of tumor residual with high sensitivity.	Chances of false negative or false positive findings. Requires expertise and skilled trainers. Low diagnostic accuracy in meningioma and metastases. Requires more time in awake craniotomy which leads to pulmonary embolism, and venous thrombosis. Rate of complete resection of tumors is lower with iMRI alone.
Intraoperative Fluoroscopy [81-86]	Facilitates tumor delineation. Maximizes the extent of high-grade glioma resection. Causes the brain tumor to lighten, and increasing accuracy. Safe, useful and universally available. Enhances blood-brain barrier rupture.	Subjective colour discrimination may cause problems. Specificity is low. Under dark field conditions, visualization of anatomical landmarks is compromised. Some of the brain-harboring glioma may not give fluorescence. Bleeding cannot be addressed in dark field conditions.
Intra-operative computed tomography (iCT) [25-28]	Provides better bony resolution. Decrease radiation exposure. Helps in detecting brain shift with high accuracy. Helpful in biopsy of small target volume tumors.	High risk due to radiation. The modality is expensive. Movement of large machine in and out causes high risk of infections. Improper positioning of the patient may cause alteration of image quality.
Functional magnetic resonance imaging (fMRI) [50-61]	Critical for pre-surgical planning, mapping brain functions. Task-based fMRI aids in detecting motor and language areas. Resting-state fMRI allows multiple brain function assessment without patient cooperation.	Task-based fMRI requires patient cooperation, challenging for certain patients. Lower specificity in high-grade gliomas. Limitations in data processing for resting state-fMRI.
Positron emission tomography (PET) [62-70]	High sensitivity in tumor detection, especially with amino acid-based tracers. Differentiates scar tissue from active tumors. Effective in detecting gliomas and meningiomas. Useful in assessing treatment responses and tumor metabolism.	Poor image resolution. Exposure to high levels of radiation. Interference from normal brain activity in fluorodeoxyglucose (FDG) PET. High costs and limited availability.
Magnetic resonance spectroscopy (MRS) [72-80]	Non-invasive, provides insight into tumor metabolism. Effective in distinguishing tumor grades and types. Useful for assessing residual tumors intraoperatively. Enhances accuracy when combined with other modalities like diffusion-weighted imaging.	Lower sensitivity and specificity compared to some PET modalities. Limited clinical adoption of ultra-high field 7T MRS. Can be less reliable without a combination with other imaging modalities.
Endoscopy [93-100]	Minimally invasive nature. Visualization is enhanced. Reduced surgical morbidity. Shorter hospital stays. Improved patient recovery.	Difficult learning curve for operators and surgeons. Specialized equipment needed. Limited range of motion. 3-Dimensional visualization is not available. Challenging ergonomics and fatigue. Careful patient selection.
Intra-operative ultrasound (IOUS) [30-49]	Less expensive than other modalities. Does not prolong surgery time. Low-grade gliomas are better defined with IOUS than CT/MRI. Shows significant definition of brain tumors in comparison with intra-operative CT/MRI. Better diagnostic precision in diagnosing residual tumor than iMRI.	Challenging interpretation than intra-operative MRI/CT. IOUS is prone to artifacts. Steep learning curve of IOUS. Does not provide synoptic view of the brain, and the image quality is highly variable and operator-dependent. Restricted to the craniotomy, providing a limited field of view.
Single photon emission computed tomography (SPECT) [87-92]	It is less expensive than CT/MRI. SPECT is widely available. Detects early changes in metabolism that lead to early tumor detection. Helps in detecting tumor progression. It is a non-invasive modality.	Lower special resolution than PET and MRI. Longer scanning time from PET and MRI. Causes exposure to radiation. Artifact-prone. Less sensitive than MRI. Less anatomical detail as compared to CT/MRI.

Surgical procedures for tumor resection

Awake Craniotomy

The aim to increase the quality of life of patients after treatment of brain tumors has led to an exploration of techniques that aid in improving intraoperative assessment of neurological status and minimizing neurological deficits. The only method that can provide an assessment of all eloquent areas of the cerebral cortex and white matter is brain mapping during awake craniotomy [101] which otherwise is unattainable under general anesthesia (GA). The specialty of awake craniotomy (AC) is its ability to analyze eloquent brain areas making it a valuable method for reducing the risks associated with tumor resection, especially concerning motor and language components [102]. AC not only ensures that the patient has a good quality of life but also provides great neuro-oncological result. Apart from the medical aspects of awake surgery, its economic issues are also favorable [101]. AC tumor surgery has reduced the morbidity associated with brain tumor resection, allowing patients to mobilize and be discharged earlier [103].

A study conducted by Brown et al. compared craniotomy for tumor resection under GA and AC. Data collected was analysed for the length of hospital stay (LOS), the extent of resection (EOR), operating time and post-surgical neurological sequelae. The study included a total of eight studies with 951 patients (411 utilizing AC and 540 utilizing GA) were included. The interpretation is that AC (4 d, n=110) results in a shorter LOS compared to the GA (9 d, n=116). The mean EOR was slightly lower under awake conditions (41%, n=321) versus GA (44%, n=444), and postoperative deficits or complications were less under AC (7%, n=411) versus GA (23%, n=520). Surgery time was slightly less in the AC group (165 min, n=324) versus GA (168 min, n=477) [104].

However, in a prospective randomized comparative study conducted by Gupta et al., the results of surgery under AC versus surgery under GA for intrinsic eloquent area lesions were compared, which included 53 patients with brain tumor in the eloquent areas who were prospectively randomized (26 patients in AC group and 27 for surgery under GA). At three months follow-up it was found that 23% of patients in the AC group had permanent deficits compared to 14.8% in the GA group. However, more than 90% tumor excision was achieved in 57% of patients in the AC group compared to 73.7% in the GA group. The study concluded that the blood loss and mean operative time were found to be less in the GA group patients compared to the AC group. Better tumor reduction and neurological improvement were seen in the GA group (speech improvement in 62.5% and motor improvement in 35.7%) than in the AC group (speech improvement in 14.3% and motor improvement in 18.7%) [105].

Another study conducted by Sacko et al. prospectively compared two groups of patients who underwent surgery for supratentorial lesions. One group underwent awake craniotomy with intraoperative brain mapping (AC group, n = 214) and another under general anaesthesia (GA group, n = 361, this included 72 patients with lesions in eloquent areas). The AC group included lesions near the eloquent cortex that were surgically treated on an elective basis. The two groups were almost similar in terms of gender, age, pathology, tumor size, EOR, duration of surgery, American Society of Anaesthesiologists score, and neurological outcome, and different in tumor location and preoperative neurological deficits, which was higher in the AC group. However, it was seen that patients with lesions in eloquent areas showed better neurological outcomes and EOR (P < .001) in the AC group than the GA patients with lesions in eloquent areas. The surgery was uneventful in AC patients and they were discharged earlier [106].

A systematic review and meta-analysis were conducted by Sattari et al. to compare AC and craniotomy under GA for the resection of gliomas in the eloquent regions. Primary outcomes were overall survival, progression-free survival, the extent of resection (EOR), neurological deficit, Karnofsky performance score (KPS), and seizure-free period at the three-month follow-up. Secondary outcomes included the duration of surgery and LOS. A total of 800 patients (39.4%) underwent AC and 1232 (60.6%) underwent asleep craniotomy. The meta-analysis concluded that the AC group had overall survival (MD = 2.86 months [1.35, 4.37], P = .0002), better EOR (mean difference [MD] = MD = 8.52 [4.28, 12.76], P < .00001), progression-free survival (MD = 5.69 months [0.75, 10.64], P = .02), three-month follow-up KPS (MD = 13.59 [11.08, 16.09], P < .00001), and at three-month follow-up seizure-free period (odds ratio = 8.72 [3.39, 22.39]) (P < .00001). In addition to that, the AC group had a lower three-month postoperative neurological deficit (odds ratio = 0.47 [0.28, 0.78], P = .004) and LOS (MD = −2.99 days [−5.09, −0.88], P = .005), but the duration of surgery was similar between the groups (MD = 37.88 minutes [−34.09, 109.86], P = .30) [107].

A multicenter retrospective cohort study was conducted by Chowdhury et al. which compared patients who underwent high-grade glioma resection by AC method and those who underwent craniotomy with general anesthesia (GA). A total of 891 patients were included, out of which 79% were subjected to GA, and 21% underwent AC. There were no differences in the median progression-free survival between awake craniotomy (0.54, 95% confidence interval [CI]: 0.45-0.65 y) and GA (0.53, 95% CI: 0.48-0.60 y) groups (hazard ratio 1.05; P<0.553). The median overall survival was longer in the AC group (1.70, 95% CI: 1.30-2.32 y) compared to the GA (1.25, 95% CI: 1.15-1.37 y) group (hazard ratio 0.76; P<0.009) but this effect did not remain after controlling for other variables. The median LOS was shorter in the AC group (2 [range: 0 to 76], interquartile range 3 d vs. 5 [0 to 98], interquartile range 5d for GA groups, P<0.001). Pain scores showed similar results between the groups [108].

Many factors are to be considered while comparing like complications, tumor resection percentage, re-admission rates, complications and post-operative outcome. Given the effectiveness of AC for resection of eloquent tumors, the data suggests an expanded role for AC in brain tumor surgery regardless of tumor location [104]. Awake craniotomy for tumors in the eloquent regions has shown better EOR, overall survival, lower postoperative neurofunctional deficits, and shorter LOS. When feasible, the authors recommend awake craniotomy for surgical resection of gliomas in the eloquent regions [107]. Awake craniotomy with brain mapping is safer and more effective in the removal of lesions close to functional areas with fewer neurological complications. Hence, it is an excellent alternative to craniotomy under GA [106].

A retrospective study conducted by Groshev et al. included a total of 76 brain tumor patients. Resected tumors included glioblastoma (34%), WHO grade III anaplastic astrocytoma (18%), metastasis to the brain (41%), WHO grade II glioma (4%), WHO grade I glioma (1%), and meningioma (1%). More than half of the procedures were performed in the frontal lobes, followed by the temporal lobe, and occipital lobe. The common indications were motor cortex and primary somatosensory area lesions followed by speech area lesions. The EOR was gross total for 59% of the patients, near-gross total for 34%, and subtotal for 7% of the patients. The average LOS for the cohort was 1.7 days with 75% of the patients staying at the hospital for only 24 hrs or less following the surgery. In the postoperative period, 21% of patients experienced no change, 67% of patients experienced an improvement in neurological status, 7% of them experienced transient neurological deficits, which were later resolved within a period of two months post-op, 1% experienced transient speech deficit, and 3% of them experienced permanent deficits. The study concluded that AC is a good choice for maximum-safe resection for primary and metastatic brain tumors and is associated with a short LOS and lower postoperative complications [109].

AC is rapidly becoming the popular method for resection of brain tumors occurring in eloquent areas. However, it is estimated that about 30% of patients undergoing AC can experience intraoperative seizures (IOS). Although IOS is one of the most common operative complications of AC, the neurological deficits and consequences of this complication have not been studied in detail. IOS can have an adverse effect on intra-operative monitoring, and surgical performance, resulting in increased operative time, may necessitate conversion to general anaesthesia, more extended hospital stays, or inadequate extent of tumor resection, increased postoperative morbidity and poor survival. A study conducted by Shah et al. investigated possible predictors of IOS during AC for brain tumor resection, including seizure history, tumor location, patient demographics, and antiepileptic use. In this study, seven (3.5%) of the 200 patients who underwent AC experienced IOS. However, none of the ACs were converted to GA due to AC failure, and there was no mortality recorded. It was found that the tumor location has been strongly associated with IOS during AC, especially the frontal lobe involvement has been reported to be associated with a higher risk of IOS, along with the supplementary motor area (SMA) and Rolandic area. They did not find a significant correlation between the incidence of IOS and preoperative seizure history. The study concluded that a relatively low percentage of people experience IOS during AC and need to evaluate other variables to help minimize the incidence of IOS [110].

Patient experience: One of the important factors for a surgery to become popular and widely accepted is the patient experience. Patient experience includes pre-operative, operative and post-operative experience in terms of pain, stress, outcome and psychological impact. A study conducted by Starowicz-Filip et al. included 18 brain tumor patients. The Essener Trauma-Inventory Questionnaire and the Addenbrooke's Cognitive Examination (ACE III) were used for evaluation. The patient’s experience with AC was done with a qualitative descriptive survey. The study showed that all patients remembered the intraoperative neurological examination and several sensations including head clamp fixation or having eyes covered, drilling, cold so on. In most of the patients, the postoperative psychological trauma experience did not reach the clinical level requiring treatment. The ACE III scores showed partial cognitive deficits with low scores in word fluency and memory. Slight amnestic aphasia was observed in two patients. The study concluded that AC is well-tolerated by patients and does not cause significant psychological trauma [111].

Another study conducted by Fontaine et al. dwelled on the postoperative perception of the awake craniotomy procedure and showed that about half of the patients have experienced some degree of intraoperative pain. Pain was mild in intensity between 1 and 2 on the visual analogical score, mostly short-lasting, and did not cause any difficulty in the procedure. Pain was reported as moderate in about 25% of patients and exceptionally severe in rest. For neurosurgeons, the most challenging causes of intraoperative pain were inadequate installation, and the contact of surgical tools with pain-sensitive structures like the dura mater of the skull base, falx cerebri, and the leptomeninges. The study concluded that these problems can be reduced by focusing the patient on the functional tests to distract their attention away from the pain and avoiding the pain-sensitive structures during the awake phase of surgery, along with preoperative patient information and preparation, presence of trained anaesthesiologists are key factors to prevent intraoperative pain and provide postoperative satisfaction [112].

Another study conducted by Tan et al., about AC as the choice of tumor resection, collected data on patient experience and its acceptance in the Asian population. Data on patient experience were collected by a structured questionnaire at the follow-up appointment. Data on patient demographics and diagnosis were collected from their respective medical records. Eighteen patients (aged 16-68 years) who underwent 20 ACs were included in the study. Preoperatively, all (100%) patients understood the indication for the surgery. Almost all patients felt fully counselled by the anaesthetist (100%), neuropsychologist (95%) and neurosurgeon (90%); 95% reported that their family was supportive for AC and 75% felt prepared on the operation day. During the operation, most patients did not experience any pain/discomfort (55%) or anxiety (65%). Most of the patients said intraoperative motor (100%) and language testing (90%) was easy. Postoperatively, 100% of them were satisfied with their care; 100% of them said their overall experience was good or excellent and 85% were willing to undergo AC again if needed. The study concluded that AC is well-accepted in the Asian population and all patients had good-to-excellent overall experience [113].

Outcomes and complications: Functional preservation or improvement following a surgery is a keystone in neurosurgical tumor resection, although collateral damage may be unavoidable in certain situations and also depends on the area and extent of tumor spread. The surgeons always aim to achieve a high percentage of tumor resection with the best possible neurological sequelae. A study conducted by Akay and Islekel reported the morbidity, functional outcomes and complications in patients who underwent AC. This study involved 46 cases, two of which were paediatric cases with lesions in the functional area and operated with the AC method. The average age was 48 years. Both preoperative and postoperative neurological examinations were recorded at three-month intervals. The results showed that out of the 46 patients who had AC, 17 of them had neurological deterioration in the intraoperative period and at the month 1 follow-up, 13 of these 17 patients had full neurological recovery. Four patients who developed hemiplegia were later able to mobilize with support at month 6 follow-up. All the patients had a return of language skills to baseline preoperative function at one month. When the results of the AC method were examined, it was observed that persistently the postoperative neurological dysfunctions were very less and it could be concluded that the precise knowledge of the surgeon, the anaesthesia team and the patient cooperation is required for AC [114].

However, in a study conducted by Kurian et al., they assessed all the articles published in the last 20 years describing complications of patients who had undergone either awake or asleep mapping for eloquent brain tumor resection. They analysed the number of patients, follow-up duration, cases of motor and sensory deficits, and outcomes at one-month follow-up. Nine out of 31 studies selected directly compared the outcomes of awake vs asleep mapping. The rate of transient deficits among patients who underwent awake mapping was 31.6% and asleep mapping was 32.7%. The rate of permanent deficits was seen in 10.8% of awake-mapping patients and 12.7% of asleep-mapping patients. It was concluded that the motor and sensory complications occurred at similar rates and rates of transient and permanent postoperative neurologic deficits were similar in both groups. Motor mapping with direct cortical stimulation (DCS) is useful for motor function preservation. However, many patients still experience postoperative motor dysfunction even after undergoing tumor resection following motor mapping [115].

A study conducted by Fang et al. evaluated recovery of motor function by muscle strength testing before surgery and 3, 7, 14 days, and three months after surgery. They found that half of the patients experienced transient motor impairment within a week. Six patients suffered from permanent motor deficits out of which four had type III glioma. Compared to types I and IV, patients with type III gliomas took three times longer to recover. Patients with types I and II gliomas were more prone to epilepsy than those with types IV and III gliomas. Classification of gliomas was useful in predicting postoperative motor function prognosis in patients who underwent motor mapping with direct cortical stimulation. Conservative strategy should be preferred in cases where gliomas are located in proximity to the posterior limb of the internal capsule [116].

A study conducted by Bonifazi et al. evaluated the neuropsychological and neuro-oncological outcomes of 19 patients who underwent AC for resection of malignant tumors located in eloquent areas. The study showed that, post-surgery, language functions were unchanged in 80% of patients and slight impairment in memory and executive functions was seen in about 50% of patients. The survival rate at one-year follow-up was 89%. Results showed that the awake procedure is safe, well tolerated by patients, and provides good linguistic and cognitive outcomes similar to low-grade gliomas. The majority of patients reported a good quality of life [117].

A retrospective study was conducted by Clavreul et al. to evaluate the extent of resection (EOR), and functional and survival outcomes in patients with glioblastomas (GB) following AC. A group of 46 patients with primary GB treated with the Stupp regimen was taken and assessed for EOR, progression-free survival (PFS), overall survival (OS), postoperative language and motor deficits three months following AC. The study showed that complete resection was achieved in about 61% of the 46 GB patients. The median progression-free survival was 6.8 months (CI 6.1; 9.7) and the median overall survival was 17.6 months (CI 14.8; 34.1). Three months after AC, more than half of the patients who were asymptomatic preoperatively remained asymptomatic, and one-third of patients who were symptomatic preoperatively experienced improvements in language, but not in motor functions. The risk of postoperative deficits was higher in patients who had preoperative deficits or incomplete resection. The study concluded that AC is an option for resection of GB in critical locations and the observed survival outcomes are expected for GB patients in the Stupp era. However, given the aggressiveness of GB, the success of AC in terms of the recovery or preservation of language and motor functions cannot be guaranteed [118].

Hospital stays greatly impact the financial as well as work-life balance. There is rising popularity in surgeries focusing on reducing hospital stays and mobilizing patients early and returning to their daily activities as early as possible. A study conducted by Chen et al. compared asleep under GA and awake deep brain stimulation (DBS) procedures, to assess the LOS, adverse events and 30-day readmission rates. Results showed that of 284 patients, 126 (44.4%) underwent awake surgery and 158 (55.6%) underwent asleep surgery. The most frequent overall complication was a change in mental status (13 patients), followed by haemorrhage (four patients), seizures (four patients), and hardware-related infection (three patients). The mean LOS for all 284 patients was about 1.19 ± 1.29 days (for the awake group: 1.06 ± 0.46 days; and for the asleep group: 1.30 ± 1.67 days; p = 0.08). Overall, the 30-day readmission rate was 1.4% (three asleep and one awake group patient). No significant differences in complications, LOS, and 30-day readmissions were found between awake and asleep groups [119].

Another study conducted by Ruichong Ma et al. included patients undergoing elective endoscopic (n = 65) or awake (n = 10) tumor resection, and showed that 66.7% of patients undergoing these procedures could be discharged safely within one postoperative day. Of the patients who stayed longer, 76% had longer stays because of either social reasons or failing occupational therapy assessments. Only six cases (24%) of longer hospital admissions were because of medical reasons [3]. The study concluded that an early discharge after endoscopic and awake craniotomy tumor resection is both safe and possible in most patients and is not associated with an increase in morbidity [103].

A study was conducted by Kwinta et al. to determine the frequency and consequences of intra- and postoperative adverse events for intrinsic supratentorial brain tumors following AC. Surgery-related complications were seen in 23 patients (92%) and postoperative complications were seen in 17 cases (68%). The most common surgery-related inconvenience was intraoperative hypertension (eight cases), followed by discomfort seen in seven cases, pain during surgery in five cases, and tachycardia in three cases. The most common postoperative adverse event was language deficit which occurred in 10 cases and was permanent in one case. Motor deficits occurred in 36% of cases and were permanent in 1%. Seizures appeared more often in patients with multilobar insular-involving gliomas and patients with no prophylactic antiepileptic drug administration. Seizures were observed in four cases intra-operative and two cases postoperative. The study concluded that surgery-related inconveniences and postoperative complications occur in most ACs [120].

A propensity score-matched cohort study conducted by Gerritsen et al. included patients aged 18-90 years, undergoing tumor resection, had a histopathological diagnosis of primary GB, tumor located eloquent or near-eloquent location, and had unifocal enhancing tumor. Patients either underwent AC or asleep resection, as per the surgeon or multidisciplinary tumor board decision. A total of 3919 patients were taken, of which 1047 patients had primary eloquent glioblastoma and were included in analyses. After propensity-score matching, the overall matched cohort comprised 536 patients, of whom 134 underwent AC and 402 had asleep resection. In the overall matched cohort, patients undergoing AC resulted in fewer neurological deficits as compared to patients undergoing GA at 3 months (22% vs 33%, p=0.019) and 6 months (26% vs 41%, p=0.0048) post-operatively. Following the surgery, longer OS was seen in AC (median 17.0 months [95% CI 15.0-24.0]) vs GA (14.0 months [13.0-16.0]) p=0.00054, and longer PFS for AC (median 9.0 months [8.0-11.0]) vs GA (7.3 months [6.0-8.8]; p=0.0060). Fewer postoperative neurological deficits were seen in the AC group compared to the asleep group at three months in patients aged 70 years and older (2 [13%] of 16 for AC vs 15 [43%] of 35 for GA; p=0·033) but no difference was seen at six months and at six months in those with a KPS of 80 or less (5 [18%] of 28 for AC vs 34 [39%] of 88 for GA; p=0.043) but no difference was seen at three months [121].

The above data suggests that awake craniotomy is one of the better options for brain tumor resection. Awake craniotomy not only achieves a high degree of tumor resection but also aims at preserving neurological functions. Patients who underwent awake craniotomy had good acceptance of the procedure and had good post-operative outcomes, including better preservation of motor, language, speech, better survival rate and quality of life. The hospital stays in patients undergoing AC was less compared to other traditional craniotomy and also had fewer re-admissions. However, many studies did yield equivocal results when compared to AC but in such studies, the number of patients undergoing AC was less compared to other traditional methods, also the studies clearly suggest that there is a need for further research in this area with a larger population to arrive at a much definitive conclusion. Added to that intraoperative and post-operative complications did not merely depend on the surgical technique but also on the pre-op status of the patient and risk factors. Other factors like the patient's age, type, stage and location of the tumor play a more important role in the post-surgical outcome and survival rate than just the surgical technique used. Overall awake craniotomy is one of the better options to consider whenever feasible for better tumor resection and better post-surgical sequelae.

Keyhole Craniotomy

A keyhole craniotomy is one of the surgical approaches for brain tumor resection that allows the surgeon to get a wide view through a tiny opening. This technique is commonly used for meningiomas, vestibular schwannomas, skull base tumors, and metastatic brain tumors. The main goal of keyhole cranial surgery is to maximize surgical efficiency while minimizing approach-related injury while maintaining good ability to safely perform the operation. The keyhole craniotomy approaches include supraorbital, retrosigmoid, mini-pterional, transcortical, keyhole subtemporal, pineal, and transventricular approaches. Keyhole approaches require careful preoperative planning to determine the best surgical method with careful evaluation of tumor anatomy. The endoscope is a very valuable tool in keyhole approaches to increase illumination along the surgical field and look around structures that are difficult to mobilize. The benefits of keyhole craniotomy include less pain after the procedure compared to an open craniotomy, better preservation of neurological functions, less scarring, and a more rapid recovery [122]. A study conducted by Reisch et al. covering the technical details of the supraorbital key-hole craniotomy using the frontolateral approach suggests that the supraorbital craniotomy avoids removal of the orbital rim, the lesser sphenoid wing or the zygomatic arch and also allows wide intracranial exposure of the deep-seated supra- and parasellar region. The limited craniotomy needs lesser brain retraction thus significantly decreasing approach-related morbidity. Added to that, the short skin incision within the eyebrow, single burr hole and meticulous soft tissue dissection results in a good cosmetic outcome [123].

In a study conducted by Iacoangeli et al., 56 patients were admitted for the surgical removal of anterior cranial base meningiomas. Out of which 33 patients underwent a traditional craniotomy tailored to the lesion and in other 23 patients, a minimally invasive keyhole supraorbital approach according to the Perneczky technique was used. In the first group, it was observed that there were 10 males and 23 females with a mean age of 62.5 years while in the second one, there were nine males and 14 females with a mean age of 64.5 years. Neuroimaging studies were performed to obtain details on tumor dimensions, localization of the tumor, analyze the architecture of the tumor, and also see prelesional edema and mass effect which may eventually lead to compression of vital structures. Following the surgery results showed that total tumor resection, with resection of the basal dura and drilling of the hyperostotic bone, was achieved in 27 out of 33 patients in the first group and 19 out of 23 in the second group. Postoperatively, four complications were seen in patients who underwent traditional craniotomy (12.5%) represented by three cases of cerebrospinal fluid (CSF) leakage, which was solved by positioning of an external lumbar drainage, and one case of contralateral hemiparesis caused because of an ischemic insult. On the other hand, the supraorbital keyhole approach had three postoperative problems (8%) represented by one case of CSF leakage which was solved in the same way as mentioned above, and one case of visual deterioration. For comparison tumors were divided into small (<2.5 cm), intermediate (2.5-4.5 cm) and large (>4.5 cm) based on their dimensions. They observed that most of the complications happened after the removal of small or intermediate meningioma and little to no complications were seen after the resection of larger tumors. On comparing the duration, the keyhole group took a mean duration of 5.8 hours whereas the traditional craniotomies group took a mean value of 5.03 hours. Another important parameter observed was the duration of hospital stay in patients who were subjected to the supraorbital keyhole approach. The observed mean hospitalization was five days in case of regular hospitalization and 23 days in case of postoperative complications, and a mean hospitalization of seven days in patients who underwent traditional approach without postoperative complications and 22 days in patients who experienced complications like CSF leakage or other problems [124]. However, the cosmetic outcome was better in the supraorbital keyhole group compared to the traditional craniotomy group. Out of 23 patients who underwent supraorbital keyhole craniotomy, only one patient (4.34%) had aesthetic problems and one presented with a small depression behind the temporal line, the location where burr hole trephination was done. This complication was overcome in subsequent operations by the use of piezosurgery, as piezosurgery allowed a thin and regular bone cutting aiding the bone flap to heal and preventing indentation of the overlying skin resulting in better cosmetic outcomes. The other patients never observed loss of eyebrows, and the surgical scar was well hidden by the eyebrows. In the traditional craniotomy group, there were three cases of cosmetic deformities (9.09%) due to atrophy of the temporal muscle in two cases and a complete lesion of the frontotemporal branch of the facial nerve in another case. The study concluded that the supraorbital approach is unsuitable for most lesions but the indications must be tailored depending on the patient. The biggest limitation of the keyhole approach was the problem of lighting with the operating microscope in a narrow corridor. But it has been largely overcome by endoscopy assistance which gives better visualization. The supraorbital keyhole minimally invasive approach via an eyebrow skin incision was said to be a valid alternative to the traditional craniotomies in anterior cranial fossa meningioma surgery. The keyhole approach can even be used for deep-seated lesions, this minimally invasive procedure provides the same extent of resection and chances of tumor control, as the wider craniotomies counterparts, without increasing the complications, hospital stay, and cosmetic disfigurement [124].

A study conducted by Banu et al. aimed at a comparison of endoscope-assisted endonasal versus supraorbital keyhole resection of olfactory groove meningiomas. Nineteen cases were taken and divided according to the operative technique into three different groups: purely endonasal (six cases), supraorbital eyebrow (microscopic with endoscopic assistance (seven cases)), and combined endonasal endoscopic approach with either the bi-coronal or eyebrow microscopic approach (six cases). Tumors were assessed based on the Mohr radiological classification and the presence of the lion's mane sign and the tumor resection was assessed by postoperative MRI using volumetric analysis. The mean age of patients at surgery was 61.4 years. The mean tumor volume was about 19.6 cm in the endonasal group, 33.5 cm in the supraorbital group (SO), and 37.8 cm in the combined group. The majority of tumors were either Mohr Grade II (moderate) (42.1%) or Grade III (large) (47.4%) and significant frontal lobe oedema was identified in 10 cases (52.6%). Gross-total resection was 100% in the SO cases with endoscopic assistance, 66.7% for the combined cases, and 50% for the endonasal cases. The extent of resection (EOR) was 100% for the supraorbital eyebrow cases, 98.9% for the combined cases, and 87.8% for the endonasal cases. Postoperative complication like anosmia was seen in 100% of the endonasal and combined cases and only in 57.1% of the SO cases. Excluding anosmia, permanent complications occurred in 0% of the cases in the supraorbital eyebrow group, 16.7% of cases in the combined group, and 83.3% of the cases in the endoscopic group (p = 0.017). The tumor recurrences were: two in the endonasal group and one in the combined group. The study concluded that the supraorbital key-hole approach, with endoscopic assistance, leads to a higher degree of tumor resection and a lower rate of complications compared to the purely endonasal endoscopic approach. The combined above-and-below approaches may be needed for large tumors with invasion of the ethmoid sinuses but the endonasal endoscopic approach by itself may be suitable for few cases [125].

A meta-analysis was performed comparing the endoscopic endonasal and open craniotomy approach for tuberculum sellae meningioma. There were 38 retrospective references, out of which 33 were for the open cases and eight for the endoscopic endonasal approaches and three had both approaches. Results showed a similar rate of gross total resection between approaches, about 85% for open cases and 84% for endoscopic cases. However, there was a much higher rate of cerebrospinal fluid (CSF) leak in the endoscopic cases, about 26.8% whereas just 3.5% in open cases. It was also found that usage of rigid reconstruction and/or a vascularized nasoseptal flap resulted in a 16% leak rate versus 64% for other closure methods. Rates of pituitary dysfunction were low and similar across the studies. Vision loss was significantly higher in open approaches, about 9.2% for open cases versus 1.3% for endoscopic cases, but the open cases mostly included larger tumors. Unfortunately, this comparison included multiple types of open approaches grouped. Keyhole approach through an eyebrow incision was used to resect tuberculum sellae meningiomas in 78 cases. Complications were seen in eight patients with worsening vision, one with corneal abrasion, seven with hyposmia/anosmia, five with endocrinological problems, and two patients who died. Gross total resections were achieved in 67/78 (85.9%) cases. There were also three cases of CSF leaks and no wound infections. These results were similar to the open approach showing no greater risk, with a similar rate of gross total tumor resection, despite the smaller craniotomy size. Supraorbital keyhole approach was performed to resect 81 olfactory groove meningiomas. Seventy-four tumors were resected by gross total fashion (91.4%). Complications included eight cases with CSF leaks and five with wound complications. This higher rate of CSF leak and complications could be due to the midline location of olfactory groove meningiomas and also making it difficult to visualize. However, in a recent study compared traditional open craniotomy with endoscopic endonasal resection of tumors, it was concluded that better resections and lower CSF leak rates were possible through the open rather than endoscopic approach. Overall, the use of the endoscope for visualizing the cribriform plate may further help in complete resections of olfactory groove meningiomas and also help with skull base reconstruction to prevent CSF leakage [126]. A study including 43 patients was reported with either craniopharyngiomas or anterior skull base meningiomas resected through an extended endoscopic endonasal approach or through a keyhole supraorbital craniotomy approach. The study showed that the supraorbital craniotomy approach through the eyebrow allowed access to a number of lesions in the subfrontal area with minimal brain scarring and a much smaller potential for injury of the superficial structures. Minimally invasive techniques have a learning curve, smaller and simpler lesions should be treated first before moving on to larger and more complicated brain lesions. The midline and suprasellar lesions are much more easily accessible through this approach than laterally-based lesions. This approach not only gives great cosmetic results but also has surgical efficacy and less complications. The pterional craniotomy is a very important part of cranial surgeries that provides access to the anterior and middle fossae. However, the latest “keyhole” approaches, including the micropterional or pterional keyhole craniotomy (PKC) provide great exposure for many conditions and also reduce the surgical morbidity. The PKC was found to be associated with reduced operative time, shorter hospitalizations and better cosmetic outcomes. In addition, it represents an ongoing trend toward smaller craniotomy size for cranial surgical procedures [127].

Another study was conducted by Reisch et al. on a minimally invasive supraorbital subfrontal keyhole approach. The study included cases during a five-year period between January 2003 and December 2007, which used the supraorbital approach in 21 cases for temporal mesial lesions, and in 15 cases, the lesion was located in the dominant hemisphere. In all cases, preoperative planning was done, and extension of the tumor within the parahippocampal gyrus or deep temporobasal tumor growth below the sphenoid wing was considered as exclusion criteria for using the supraorbital approach. It was observed that in all the cases, surgery was performed without any intraoperative complications. Pathological investigation showed four high-grade astrocytomas, seven low-grade astrocytomas, two cavernomas and two gangliogliomas. Postoperative MRI scans showed complete removal of the tumors in 14 cases. The postoperative neurological examination showed no change in 14 cases, one case showed a transient hemiparesis and in patients who had dominant-sided lesions, there were no speech or mental deficits. The study concluded that, in selected cases, the minimally invasive keyhole supraorbital craniotomy offers very good surgical outcome in the temporomesial region with little to no approach-related morbidity compared to a standard pterional-transsylvian or transtemporal approach [128].

A retrospective analysis was conducted by Avery et al. to compare the indications, outcomes, and anatomical limits of supraorbital (SO) and mini-pterional (MP) keyhole craniotomies in patients with intra- and extra-axial brain tumors. The data analysed included the extent of resection (EOR), length of stay (LOS), pathology and complications. Using preoperative MRI data, tumor heatmaps were made in order to compare surgical access provided by both methods. Results showed that 158 patients underwent 173 (84.8%) supraorbital craniotomies, and 30 patients underwent 31 (15.2%) MP craniotomies out of which 71 (34.8%) procedures were reoperations. Out of 204 operations, 110 (63.6%) were done by SO and 21 (67.7%) by MP approaches, for extraaxial tumors. 56.1% of the parasellar tumors were resected by the SO approach and 41.9% by the MP approach. Axial projection heatmaps indicated that supraorbital access included the parasellar region, ipsilateral Sylvian fissure, entire ipsilateral and medial contralateral anterior cranial fossa, medial middle cranial fossa, and anterior midbrain, whereas MP access was confined to the lateral parasellar region, ipsilateral middle cranial fossa, Sylvian fissure and posterior aspect of the anterior cranial fossa. Coronal projection heatmaps indicated that parasellar superior access was greater with the SO approach compared with that of the MP approach. Endoscopy was used in 98 (56.6%) SO craniotomies and seven (22.6%) MP craniotomies. Endoscope-assisted tumor removal was used when tumors were farther from the craniotomy or in angled areas such as cribriform plate where microscope usage is limited. Near total resection or gross total was seen in 120/173 (69%) SO approaches and 21/31 (68%) MP approaches. Major complications were observed in 11 cases (6.4%) SO approaches and one case (3.2%) MP approach (p = 0.49). The study concluded that both the SO and MP craniotomy approaches are versatile, safe, and complementary approaches for tumors located in the perisylvian and parasellar regions, also anterior and middle cranial fossae. The SO route which was used in 85% of cases, achieved better overall reach and versatility compared to the MP route. Both approaches benefit from added visualization with the usage of endoscopy. SO and MP craniotomies can be considered as complementary keyhole approaches for intraaxial and extraaxial brain tumor [129].

A retrospective study conducted by Lin et al. included 62 patients who underwent a total of 64 operations using the SO keyhole approach. Meningioma was the most common tumor resected, followed by pituitary adenoma and craniopharyngioma. Age, sex, tumor volume, complications and operative duration were taken into account. Pre- and postoperative residual tumor volumes were measured using OsiriX software (medical image viewer system) based on MRI. The subjects were divided into large versus small meningioma groups (15 ml cut value). The average tumor resection rate was 95.2% for meningiomas, 53.2% for pituitary adenomas and 83.9% for craniopharyngiomas. The major complication (hemiplegia and blindness) was 4.48% in all tumors. No operative-related deaths occurred. It was observed that larger meningioma groups had longer operative times and hospital stays, and greater blood loss. No significant differences in age, sex, postoperative volumes, recurrence rates or resection rates were noted between small and large meningioma groups. The study concluded that transciliary keyhole craniotomy is safer and efficacious for anterior skull base tumors, especially for meningiomas. Excellent resection results were seen even in cases of large meningiomas [130].

A retrospective analysis was conducted by Thakur et al. on critical appraisal of minimally invasive keyhole surgery for intracranial meningioma in a large case series. It included patients undergoing meningioma resection at a referral centre from 2008-2021. The surgical goal was to achieve maximum safe removal and conservative (subtotal) in case of some invasive cases. Primary outcomes included resection rates, LOS, complications and KPS. Secondary outcomes included endoscopy usage, tumor control, perioperative treatments and acute MRI FLAIR/T2 changes to assess for brain retraction injury. Out of 329 patients, keyhole approaches were used in 193 (59%) patients (mean age 59±13; 30 (15.5%) had prior surgery) who were subjected to 213 operations of which 205 were skull base locations. Approaches included: retromastoid (n = 38 (18%)), mini-pterional (n = 20 (9%)), endoscopic endonasal (n = 74 (35%)), supraorbital (n = 73 (34%)), suboccipital (n = 4 (2%)), and contralateral transfalcine (n = 4 (2%)). Gross total or near-total (>90%) resection was achieved in 125 (59%). Major complications included: CSF leak 2 (1%), meningitis 2 (1%) and permanent neurological worsening 12 (6%). There were no PEs, deep vein thrombosis, MIs or 30-day mortality. 94% were discharged to home with favourable 90-day KPS in 176 (96%) patients. In addition to that, increased FLAIR/T2 changes were noted on POD#1/2 MRI in 36/213 (17%) cases, resolving in all but 11 (5.2%). Endoscopy usage was seen in 87/139 (63%) craniotomies, facilitating tumor removal in 55%. Tumor progression occurred in 26 (13%) patients; mean follow-up was 42±36 months. The study concluded that the minimally invasive keyhole approach and endoscopic endonasal meningioma removal are associated with comparable resection rates, low complication rates, short hospitalizations and high 90-day performance scores compared to traditional skull base approach methods. It also stated subtotal removal may be appropriate for invasive/adherent meningiomas to avoid neurological deficits and other post-operative complications, even though longer follow-up is required in such cases. With careful patient selection and good experience, these approaches can be considered as worthy alternatives to traditional transcranial approaches [131].

A study conducted by Seaman et al. included a total of 32 patients who underwent 20 supraorbital and 11 endoscopic endonasal procedures. Imaging, preoperative and postoperative analysis was done following which the safety of each approach was evaluated. The mean extent of resection through a SO approach was 88.1% and that of the endoscopic endonasal approach was 57.9% (p = 0.016). Anopsia and preoperative visual acuity deficits were more frequent in the endonasal group and persisted postoperatively (visual acuity: p = 0.004; anopsia: p = 0.011). No major complications like cerebrospinal fluid (CSF) leaks or wound-related complications were identified in the SO craniotomy group, while the endonasal group had two CSF leaks needing lumbar drain placement. LOS was shorter in the SO group compared to the endonasal group (3.4 vs. 6.1 days, p < 0.001). The study concluded that anterior skull base meningiomas can be successfully managed by supraorbital or endoscopic endonasal approaches, with SO having a slight edge over the endonasal approach. Both methods can provide excellent access to tumors, safe and efficient, but patient factors and symptoms should dictate the approach selected [132].

A retrospective analysis was conducted by Zhou et al. to explore minimally invasive neurosurgery through the supraorbital keyhole approach by neuroendoscopy with respect to resection techniques, their safety, and feasibility. A total of 39 cases were included, of which 21 cases were intracranial aneurysms, nine cases were intracranial space-occupying lesions, five cases were brain trauma, three cases were CSF rhinorrhoea, and one case was of a cerebral haemorrhage. Results showed that the symptom improvement rate of intracranial space occupying lesions was 8/9 (88.9%). The length of the skin incision in all cases was about 4 cm. All patients were statistically analyzed for bone window size, LOS, preoperative neurological symptoms, and neurological symptoms obtained from follow-up after one month of surgery. The average length of the bone window of the supra-orbital (SO) eyebrow arch keyhole was 3.77 ± 0.31 cm, and the average width was 2.53 ± 0.23 cm. The average LOS was 18.77 ± 7.00 days. The average hematoma clearance rate using neuroendoscopy was 95.00% ± 1.51%. The study concluded that neuroendoscopic minimally invasive surgery through the SO eyebrow arch keyhole approach not only has less trauma, and better cosmetic result but can also enter the intracranial area including the lateral side of the cavernous sinus, anterior cranial fossa and middle fossa [133].

In a study conducted by Shahid et al. on the SO keyhole approach, a total of 19 patients (mean age 62.75 ± 12.52 years, 68% female) underwent 20 SO craniotomies. Among them, 12 (70.59%) were WHO grade 1 meningiomas, two (11.76%) were WHO grade 2 meningiomas, three (17.65%) were GBMs, one (5.88%) was a low-grade glioma and one (5.88%) was a suprasellar residual pituitary adenoma. Meningiomas were often located in planum sphenoidale, accounting for 33.33% of cases (n = 4), followed by the olfactory groove 25% (n = 3), tuberculum sellae 25% (n = 3), cavernous sinus 8.33% (n = 1), and the fronto-orbital area involving olfactory groove 8.33% (n = 1). Two patients underwent resection for GBM. Of these two, one required a second surgery for a recurrence in the fronto-orbital region, the other patient had a tumor in the third ventricle extending into the basal ganglia and midbrain and presented with hydrocephalus. One patient was subjected to SO craniotomy for a residual pituitary adenoma in the suprasellar region. Gross-total resection (GTR) or near-total resection (NTR) was seen in 14 (82.4%) of the 17 tumor cases which underwent the procedure. Subtotal resection was done in three cases, which included a GBM, a low-grade glioneuronal tumor and a cavernous meningioma with sphenoidal extension. There were no major complications observed. Minor complications included hyposmia and development of frontalis palsy which was seen in one patient each. Three patients experienced transient frontal numbness, which resolved within three months of follow-up. No patients developed a mucocele, CSF leak, sinusitis, wound infection or stroke. Preoperatively, five patients had CN I deficits, and six patients had CN II deficits. Also, one patient had CN III, CN IV, and partial CN VI palsy. Three out of five patients with CN I deficits showed improvement, whereas patients with CN I deficits worsened. Four of the six patients with CN II deficits showed improvement in symptoms. All the patients were discharged and no patients were required in hospital rehabilitation or a skilled nursing facility. The median LOS was three days. One patient was readmitted within 30 days due to obstructive hydrocephalus and resolved with ventriculoperitoneal shunt placement. Because of the aggressive nature of GBM, one patient died. Two patients showed a drop in KPS from baseline and another showed some decline due to seizures after one year of follow-up. The study concluded that SO keyhole surgery with proper patient selection can be done safely and effectively with less complication, readmission rates and less narcotic burden [134].

A study was conducted by Martín et al. on minimally invasive keyhole approach, which was used for supramaximal frontal glioma resections. The study included patients with newly diagnosed frontal gliomas who were treated using a keyhole approach with supramaximal resection (SMR). Surgeries were performed on patients both asleep and awake. Kaplan-Meier curves were used for survival analysis. Of the 790 craniotomies performed during the study period, 47 patients met the inclusion criteria. Perioperative complications were seen in five cases (10.6%). The average hospital LOS was 3.3 days. High-grade gliomas had a progression-free survival of 14.8 months and an overall survival of 23.9 months. The study concluded that the keyhole approach enabled successful SMR of frontal gliomas without additional risks. Good anatomical knowledge and precise surgical technique are important for obtaining successful resections [135].

A retrospective study was conducted by Bander et al. comparing the keyhole supraorbital approach (SOA) and larger traditional transcranial approach (TTA) for olfactory groove meningiomas (OGMs) and they analyzed clinical, radiographic, and functional quality of life (QOL) outcomes for the same. A review of 57 patients undergoing a keyhole SOA or larger traditional transcranial craniotomy for newly diagnosed OGMs was performed. The LOS, extent of resection (EOR), olfaction, radiographic volumetric analysis of postoperative edema, and QOL (Anterior Skull Base Questionnaire) were assessed. Thirty-two SOA and 25 TTA patients were included. The mean EOR was 99.1% for TTA and 98.4% for SOA, p = 0.91. The mean LOS was comparatively shorter for SOA patients (4.1 ± 2.8 days) than TTA patients (9.4 ± 11.2 days) (p = 0.002). An association between postoperative FLAIR cerebral edema and TTA (p = 0.031) was found. Olfaction was preserved or improved at similarly in both (TTA: 47% vs SOA: 43%, p = 0.99). QOL as assessed by the Anterior Skull Base Questionnaire (ASBQ) at the last follow-up and results did not differ significantly (p = 0.74). The keyhole SOA was associated with a statistically significant decrease in LOS and less postoperative edema relative to TTA [136].

The above data suggests the advantage of the keyhole approach in tumor resection. The keyhole approach is an important minimally invasive surgical technique used for tumor resection. It not only achieves a good extent of tumor resection but also preserves adjacent areas for better post-surgical neurological outcomes. There is no doubt that small approaches reduce normal tissue disruptions and brain retraction. Earlier, in the keyhole approach, it was difficult to visualize the structures through the smaller opening but this has been largely overcome by endoscopic illumination providing a better view of the tumor and its surroundings. The outcome of keyhole craniotomy not only depends on pre-operative assessment and imaging but also on the surgical skill of the operating surgeon. There are many types of keyhole approaches like supraorbital, mini-pterional, keyhole subtemporal, retrosigmoid, pineal, transventricular, and transcortical. Each has its own advantages and disadvantages, and ultimately it is the surgeon's call to opt for the best possible technique based on the patient's findings. Many studies did show the advantages of supraorbital approaches whenever feasible resulting in a reduction of hospital stay and lower incidence of CSF leaks. However, some case studies suggested that keyhole craniotomy may limit surgical freedom leading to difficulty in achieving hemostasis and maneuvering the instruments. With advancements in technology, the existing limitations may be overcome in the future. Further studies are needed to compare the keyhole approach with other newer surgical techniques in order to choose the best possible technique for each patient scenario. Although keyhole craniotomy cannot be used in all cases, using this approach whenever possible has resulted in reduced hospital stay, better preservation of neurological functions, good extent of tumor resection, and overall, less scarring and cosmetically well accepted, making it one of the better craniotomy approaches for surgical tumor resection [137].

Tubular Retractor Systems (TRS)

Tubular retractor systems (TRS) offer an advantage by minimizing the retraction pressure and reducing local brain injury while resecting deep-seated lesions and traversing white matter tracts in contrast to retractor blades which cause asymmetric tension. Minimally invasive techniques such as TRS have been shown to decrease perioperative complications like seizure, cerebral edema and venous infarction. They require shorter operative time which in turn reduces the blood loss. They have also been shown to have an accelerated post-operative recovery when compared to open surgery [138-140].

A study published in 2020 analyzing the outcomes of transcortical transtubular resection - all performed at the University of Miami between 2015 and 2017 by a single surgeon - included a total of 112 patients presenting with an array of different tumors, the most common of which were metastatic tumors, GBM and colloid cyst which showed that postoperative complications occurred in 15.2% cases with 3.6% cases suffering permanent complications after one week of follow-up which included aphasia, memory loss, left-sided weakness, and new onset long-term seizure. The mean post-operative stay was 3.8 days and the follow-up average was 17.9 months. The mortality rate was 15.2% which was due to the progression of the disease or due to non-surgical complications [138].

Tubular retractors can be used for the resection of a variety of lesions, including subcortical, neoplastic, cystic, infectious and vascular which include colloid cysts, metastatic lesions, glioblastomas, low-grade glioma, hemangiomas, lymphoma, neurocytomas, craniopharyngioma, radiation necrosis, and pituitary adenomas [139]. Different tubular retractors are available which include modified retractors, BrainPath, ViewSite brain access system (VBAS), and minimal exposure tubular retractor (METRx) but current studies do not show that one is superior to the other [139, 140]. The tubular retractor system offers another advantage of having a complete absence of post-operative central nervous system infections that could be attributed to the limited area of the brain exposed compared to conventional surgeries [140]. Retractor-less endoscopic surgery is another minimally invasive method that is becoming increasingly common. However, it has certain limitations which include limited visualization and lack of manual manipulation by a single surgeon. Studies analyzing the usage of Vycor, Brainpath and Viewsite devices show that tubular retractors can be used as a useful adjunct for glioma surgeries as they allow bimanual operative procedures with minimal complications which include hemiparesis and visual deficits [141].

Tubular retractor usage reduces the usage of auxiliary instruments thereby decreasing the cost of the procedure [142]. Transtubular resection of intracranial tumors shows a good rate of GTR with some incidence of early complications which included confusion, short-term memory difficulties, seizures, meningitis, and motor and visual deficits. Minimal incidence of permanent complications has also been noticed which include aphasia, hemiparesis and long-term seizures [143]. Other postsurgical complications such as hydrocephalus and CSF fistula have also been observed [144]. Overall TRS offers a minimally invasive way for resecting tumors of various pathologies with minimal postoperative complications and scope for gross total resection of the tumor.

Endoscopic Endonasal Approach

A study analyzing resection of the medial wall of cavernous sinus via endoscopic endonasal approach (EEA) suggested that this technique has advantages while dealing with an endocrinology active tumor however it requires advanced surgical skills, extensive anatomical knowledge and experience in order to avoid complications. Imaging techniques such as Doppler-guided intraoperative imaging, surgical navigation system and usage of blunt-tip knives to dissect the tumor plane are recommended [145]. EEA has been shown to be inferior in the management of olfactory groove meningiomas compared to other techniques showing higher rates of CSF leaks and extra-neurological complications [146]. In the treatment of skull base meningiomas, although EEA showed a lower extent of resection rates, it provides better visualization and direct access to the lesion without causing much damage to the brain, thereby making it possible to achieve maximal safe resection [147]. A study analyzing the surgical outcomes of resection of olfactory groove meningiomas using the transbasal approach, EEA and combined endoscope-assisted transbasal approach showed that the role of EEA was limited to small tumors while the transbasal approach provided the best postoperative outcomes and lower rate of complications while dealing with larger and smaller tumors (>/< 40 mm) with intact olfaction. EEA could be considered when olfaction is already absent and as an adjunct when combined with a transbasal approach [148].

A study analyzing the outcomes of endoscopic and the traditional microscopic approach for transpituitary surgery showed that the differences in gross total resection (GTR), CSF leak, diabetes insipidus (DI), meningitis, visual, improved impairment, SIADH, new onset hypopituitarism and hypothyroidism between the two approaches were not statistically significant. However, the hospital stay was observed to be longer with the traditional microscopic transsphenoidal surgery (MTS) compared to EEA [149]. A study comparing the post-operative outcomes of the transcranial approach (TCA) and endonasal approach (EEA) for the resection of craniopharyngioma showed that TCA had a higher incidence of visual deterioration. EEA showed a significantly higher incidence of CSF leakage. However, a higher rate of GTR was observed with EEA. There was no significant difference in meningitis hypopituitarism and DI between the two approaches [150]. A similar study, however, showed a higher incidence of visual disturbance, headaches and hypopituitarism in EEA than TCA with EEA showing a lower incidence of diabetes insipidus [151].

An Italian study, including 84 patients with infradiaphragmatic craniopharyngiomas, showed that the endoscopic endonasal approach provided a direct route and a better way to manage lesions extending up to the third ventricle without breaching the diaphragm, a higher rate of GTR and satisfactory postoperative results. The most common postoperative complication observed was CSF leak. Endocrine, visual and hypothalamic disturbances were among the other postoperative complications noticed [152]. The endoscopic endonasal approach has also proved to be an effective method of intraconal intraorbital tumor resection. A retrospective study, including 20 patients managed by a single skull base team, showed that EEA is a safe approach which requires no orbital reconstruction [153]. Another study, analyzing the outcomes of EEA and TCA for treating tuberculum sellae meningiomas, showed that the GTR was marginally higher with TCA than EEA, however, surgical visual improvement was significantly higher in EEA compared to TCA with fewer complications [154]. EEA has the additional advantage of being more cost-effective compared to TCA [155].

Stereotactic Radiosurgery (SRS)

Stereotactic radiosurgery (SRS), such as Gamma Knife, is often used to treat benign and malignant brain tumors, including meningioma, paraganglioma, hemangioblastomas, craniopharyngiomas and brain metastases. SRS is an increasingly valuable tool used by neurosurgeons and radiation oncologists and has been widely used over the past decade [156]. The process involves delivering precisely targeted radiation to specific areas of the brain using 3-dimensional computed imaging [157]. SRS is primarily used for brain metastases and primary tumors which are too small to be surgically resected or it is used in adjunct with surgical resection to decrease recurrence chances.

SRS is hypothesized to provide better local control, fewer side effects and better overall survival rates in comparison to other relevant therapies. A study which looked at 1446 brain metastases (BSM) patients treated with SRS found that local control was 86% at year 1 and objective response rate was 59%. Additionally, deaths from BSM progression following SRS were rare at 2.7% and the rate of treatment-related toxic effects was 2.4%. SRS offered improved outcomes when compared to targeted or immunotherapy in BSM which showed a wide objective response rate ranging from 17-56% [158].

Another study which compared outcomes between “surgical resection” and “SRS alone” in the treatment of brain metastases found no significant difference in overall survival rates and saw a greater local tumor recurrence rate in surgical resections done without SRS. Multiple studies provide evidence for the efficacy of SRS and its indication in the management of brain metastasis. However, the literature on SRS efficacy on primary brain tumors is limited and more research is warranted [159].

Whole-brain radiotherapy (WBRT) is a commonly used practice in the management of brain metastases and is used in adjunct with surgical interventions to improve patient outcomes. However, since SRS can isolate metastatic areas, it offers increased local control and better long-term outcomes [160]. A study by Brown et al. which randomly assigned 194 brain metastases (BM) patients to either WBRT or SRS noted lesser cognitive deterioration and fewer adverse events in the SRS cohort [161]. Median cognitive-deterioration-free survival was longer after SRS than after WBRT. Several studies have also compared overall survival rates between supportive care and WBRT and have found no significant difference [162]. So, there is a shifting paradigm from WBRT to localized therapy, and treatment guidelines are changing to reflect the same. At the same time, multiple studies have also reported no significant increase in overall survival rates or distant control when SRS is used [163]. The current literature on WBRT vs SRS offers mixed conclusions and there is still a lack of high-level evidence making recommendations for the clear indication of recommending one procedure over another.

Current literature also demarcates the difference between pre-operative and post-operative results of SRS. Most research identifies post-operative SRS as an established standard of care. Although research on pre-operative SRS is still emerging. A trial comparing pre-operative SRS to post-operative SRS for patients with surgically resectable brain metastases found that pre-operative SRS had lower rates of leptomeningeal recurrence and radiation necrosis in comparison to pre-operative. Pre-operative allows for surgical resection of any irradiated normal tissue which attenuates injury tissue and reduces cytokine concentrations which catalyze radiation necrosis. Additionally, a pre-operative approach improves target delineation and systemic control. There is inadequate research on the indications of pre-operative SRS on brain tumours as few trials have been conducted. However, pre-operative SRS has shown to be an effective and safe procedure in other tumors of the body with decreased surgical complications and decreased recurrence rates [164, 165].

Consideration of radiation dosage is an important factor in SRS outcomes as increased dosage can irradiate neighboring normal tissue and increase the likelihood of side effects while inadequate dosage will increase the chances of local tumor recurrence. Fractionated SRS divides the total dose into smaller doses delivered in multiple treatments. It’s understood that necrosis and pseudo-progression can complicate local control assessment. Considering this, a study by Redmond et al. looked at SRS dosage and its effects on local control rates: for tumors ≤20 mm, single-fraction doses of 18 and 24 Gy corresponded with >85% and 95% 1-year LC rates and for tumors 21 to 30 mm, an 18 Gy single-fraction dose was associated with 75% LC rates. However, it’s important to note that all patients considered in this study had received prior cranial radiation to median doses of 30 Gy for BSM and 60 Gy for primary brain tumor patients [166].

The study also recommended fractionated SRS for larger lesions. Another recent study by Gruber et al. noted that fractionated SRS can decrease the likelihood of radiation necrosis and improve local control [167]. Another dose-effect relation study established schedules of 24 Gy, 18 Gy and 15 Gy for tumors of size smaller than or equal to 20 mm, 21-30 mm and 31-40 mm, respectively. Additionally, they noted differences in fractionation-related radio-necrosis outcomes of brain metastases and found decreased radio-necrosis levels in 5-fractions SRS when compared to a single dose or 3-fractions [168]. The authors also pointed out the lack of trials comparing radiosurgery to fractionated stereotactic irradiation.

Delivery of SRS can also be modulated by the frame used during the procedure. Frame-based procedures immobilize the skull by screws or pins while mask-based procedures use thermoplastic masks and imaging guidance to deliver the precise dosage. A study by Pichardo-Rojas et al. compared outcomes between the two models. They included data from 509 patients and concluded that RN, local tumor control and treatment time numbers showed no significant difference but more research is required in this area [169].

SRS is an effective tool in the management of brain tumors but therapy timing, dose and combination with systemic therapy are important considerations. Current literature provides insights into the efficacy and benefits of SRS - in comparison to WBRT and surgical interventions only. However, more research is warranted on pre-operative vs post-operative therapy and dosage control. Table 2 summarizes the favorable outcomes and complications of all surgical procedures.

**Table 2:** Summary of outcomes of surgical procedures GTR: Gross total resection, TCA: Transcranial approach, DI: Diabetes insipidus, CSF: Cerebrospinal fluid

Surgical Procedure	Types of Tumors Resected	Favorable Outcomes	Complication
Endoscopic Endonasal Approach [145-155]	Endocrinologically active tumors, olfactory groove meningiomas, skull base, pituitary tumors, craniopharyngioma, intraconal intraorbital, tuberculum sellae meningiomas.	Better visualization and direct access to the lesion without causing much damage to the brain. Less invasive higher rate of GTR, shorter hospital stays for lower incidence of visual deterioration than TCA. Lower incidence of DI. Higher rate of visual improvement.	CSF leaks and extra-neurological complications. Higher incidence of headaches and hypopituitarism.
Tubular Retractor System [138-144]	Subcortical, neoplastic, cystic, infectious and vascular lesions which include colloids, metastatic lesions, glioblastomas, low-grade glioma, hemangiomas, lymphoma, neurocytomas, craniopharyngioma, radiation necrosis tumor, pituitary, and adenoma.	Minimizing the retraction pressure and reducing local brain injury. Good GTR, shorter operative time, reduced blood loss, accelerated post-operative recovery, complete absence of post-operative central nervous system infections, decreased perioperative complications like seizure, cerebral edema and venous infarction.	Limited visualization and lack of manual manipulation. Early complications: confusion, short-term memory difficulties, seizures, meningitis, motor and visual deficits. Permanent complications: aphasia, hemiparesis and long-term seizures. Hydrocephalus and CSF fistula.
Stereotactic Radiosurgery [156-169]	Meningioma, paraganglioma, hemangioblastoma, craniopharyngioma and brain metastases.	Increased local control and better long-term outcomes lesser cognitive deterioration.	Increased dosage can irradiate neighboring normal tissue, inadequate dosage will increase the chances of local tumor recurrence.
Awake Craniotomy [101-121]	Gliomas, meningiomas and metastatic brain tumors.	Better preservation of neurological functions, near-total to total extent of GTR, while preserving neurological functions, better suited when excising tumors from eloquent areas, better survival rates and fewer readmissions, reduced hospital stays with early mobilization of the patient.	Dependent on imaging modalities. Blood loss may be comparable or greater than that of traditional surgeries. May have chances of intra-operative epilepsies. Require advanced technologies which may not be available at all facilities. Does not provide any cosmetic advantage. May not be tolerated by all or uncooperative patients.
Keyhole Craniotomy [122-137]	Glioblastoma, meningioma, craniopharyngioma, schwannoma and metastatic brain tumors.	Lesser tissue damage and brain retraction. Better preservation of neurological functions, good visualization of tumor and its surroundings resulting in good GTR. Can even access deeper areas with minimal injury to the surrounding structure, less scaring compared to other techniques, great cosmetic outcomes. Reduced hospital stays and shorter recovery period.	Difficulty in illumination and accessing certain areas because of small opening. Difficult to maneuver instruments and achieve hemostasis due to the smaller surgical aperture. Higher incidence of CSF leaks and hyposmia in certain cases. Require advanced instruments which may not be available at all facilities. Can be used in selective cases only. Has a greater learning curve and difficult to perform.

Role of artificial intelligence (AI), virtual reality (VR) and robotics in brain tumor surgery

The integration of augmented reality (AR), virtual reality (VR), robotic technologies and artificial intelligence (AI) in neurosurgery is transforming brain tumor resection procedures by enhancing visualization, precision of the procedure and surgical outcomes. These new approaches provide immersive preoperative planning and real-time intraoperative guidance in order to improve postoperative results. In particular, these improvements are relevant for the most complex brain tumor removal interventions [170, 171].

Notably, AR and VR technologies have found valuable applications across various neurosurgical subspecialties, significantly contributing to different stages of surgical care. These technologies have been especially useful in preoperative training and planning, allowing surgeons to visualize complex brain structures and practice surgical techniques in a simulated environment before performing the actual procedure. This immersive preparation can lead to better surgical outcomes by improving the surgeon’s familiarity with the patient's specific anatomy and the planned procedure. During the surgery itself, AR and VR assist in surgical decision-making and intraoperative workflow by providing real-time, enhanced visualizations of the brain's anatomy. This enables the surgeon to navigate intricate areas with greater precision and confidence. Additionally, these technologies help minimize risks by offering continuous, real-time feedback and monitoring of the surgical site, thus reducing the chances of damaging critical areas of the brain. One specific application is intraoperative brain mapping which involves the use of direct electrical stimulation to identify and preserve essential neurological functions during surgery. In procedures like awake craniotomy (AC), where the patient remains conscious, AR and VR can assist in mapping out cortical and subcortical regions of the brain in real-time. This is crucial in surgeries involving eloquent areas of the brain regions responsible for vital functions such as language, motor control, or vision. By monitoring the patient’s neurological functions while they perform specific tasks during the procedure, surgeons can ensure that these critical areas are not harmed, thereby minimizing the risk of permanent neurological deficits [172].

Therefore, robotics offers significant benefits for patients by increasing the precision and safety of neurosurgical procedures. These systems allow for highly accurate tumor resections, minimizing damage to surrounding healthy brain tissue and reducing the risk of complications. The enhanced dexterity and control provided by robotic systems enable surgeons to perform minimally invasive procedures, which can lead to smaller incisions, reduced pain, and faster recovery times for patients. Furthermore, the stability and consistency offered by robotic assistance reduce the likelihood of human error, contributing to better overall surgical outcomes and a lower incidence of postoperative complications. As a result, patients benefit from not only more effective tumor removal but also a quicker return to daily activities and an improved quality of life. AI can also positively impact various stages of surgery, including diagnosis, disease assessment, and procedural planning [173].

Augmented Reality in Neurosurgery

Augmented reality (AR) is significantly transforming neurosurgery by enhancing both surgical precision and patient outcomes. In the context of brain tumor surgery, Ammirati has shown that using the microscope focal point as a virtual pointer integrates AR into the surgical workflow, reducing the need for time-consuming adjustments and maintaining continuous focus on the surgical site. This approach improves efficiency and precision, particularly when dealing with deep-seated tumors where accurate navigation is crucial [170]. The AR system aids in better visualization of tumor borders and eloquent areas, potentially leading to more complete resections with minimal impact on surrounding healthy tissue.

In awake craniotomy, AR and virtual reality (VR) applications have demonstrated substantial benefits in both surgical procedures and patient outcomes. According to Mofatteh et al., VR has been effectively used to map complex cognitive functions, such as vision and social cognition, which enhances the ability to perform precise intraoperative mapping and reduce the risk of postoperative deficits. For instance, VR applications have been employed to evaluate visual field impairments and social cognition, providing real-time feedback that helps tailor surgical interventions to individual patient needs [171].

AR has also been utilized for preoperative training and intraoperative navigation, which improves surgical accuracy and potentially reduces operative time. Studies reviewed by Mofatteh et al. indicate that patients undergoing surgeries with AR-guided techniques report higher satisfaction due to better-preserved brain function and fewer postoperative complications. For example, AR-enhanced navigation can help minimize damage to critical brain areas, leading to improved functional outcomes and a reduction in the incidence of postoperative deficits. However, challenges such as incomplete visibility of important anatomical structures and the need for more user-friendly AR setups have been noted. Despite these issues, the overall impact of AR and VR on patient outcomes has been positive. Both technologies have been associated with increased surgical precision, reduced operative times, and better postoperative functional results [174]. Future advancements and refinements in AR and VR technologies are expected to further enhance their effectiveness and integration into neurosurgical practice, ultimately leading to improved patient outcomes and satisfaction.

Virtual Reality in Neurosurgery: Planning and Training

Virtual reality (VR) is increasingly transforming neurosurgery, enhancing both training and intraoperative practices. Immersive VR (IVR) allows surgeons to practice complex procedures in a risk-free environment, improving psychomotor skills and spatial understanding of patient-specific anatomy. Studies show that VR can reduce the learning curve and improve operative precision, leading to better clinical outcomes. For instance, one study demonstrated that VR-based training improved surgical accuracy by 32% and decreased procedural time by 27%, highlighting its effectiveness in neurosurgical education. In a safety study involving 30 patients undergoing awake brain surgery, VR was successfully used to map eloquent language areas while identifying additional non-eloquent regions in three patients. Notably, no severe side effects such as vertigo or VR-induced sickness were reported, although seizures occurred in nine patients, which were managed without long-term complications [170, 171, 175].

Further expanding its role, VR technology has been used to map deeper cognitive functions like social cognition and emotional recognition. During surgery, VR-based systems provided real-time cognitive assessments in 90% of patients, enabling surgeons to preserve critical brain functions and improve surgical precision [176]. When assessing surgical expertise, a study with 115 participants revealed that experienced neurosurgeons demonstrated significantly less tremor in the 8-12 Hz range and applied lower force during VR simulations compared to novices. This quantitative data underscores VR's ability to objectively measure and improve fine motor skills critical for high-stakes procedures [177]. Although long-term outcomes such as survival rates or postoperative complications directly linked to VR are not extensively studied, VR-assisted surgeries have shown promising results in enhancing spatial visualization of critical structures, potentially reducing surgical invasiveness and improving recovery times [178, 179]. Furthermore, VR accurately classified visual fields in 90% of intraoperative assessments, further supporting its potential for patient safety and functional preservation [180].

This research combined VR with ocular tracking to evaluate visuospatial and social functions during awake brain surgeries. The prospective study demonstrated that real-time tracking of patients' eye movements allowed for precise mapping of brain regions involved in these functions. In over 85% of cases, the ocular tracking system accurately assessed cognitive responses within the allowable timeframe for direct electrical stimulation (DES). Moreover, brain areas responsible for recognizing facial emotions and eye contact were successfully identified in 78% of patients, offering valuable insights that enhanced intraoperative decision-making. This innovation not only proved feasible but also contributed to reducing risks of cognitive impairment after surgery. As these studies suggest, VR continues to evolve as a critical tool in neurosurgery, improving both surgical training and patient outcomes through real-time cognitive assessments and precision-guided procedures [181].

For instance, traditional brain mapping using awake craniotomy (AC) is primarily focused on language and motor areas, but VR offers the potential to map more complex cognitive functions such as vision and social cognition, broadening the scope of intraoperative brain mapping [175]. However, despite its promising applications, VR presents certain challenges. Some patients may experience side effects like nausea and headaches, which could limit its usage. Identifying patients with risk factors for these adverse effects is crucial to ensure VR's safe and effective integration into neurosurgical practice [179].

Robotic Systems and Their Role in Neurosurgery

The application of robotic technologies in brain tumor resection has emerged as a transformative approach in neurosurgery, offering significant advancements in precision and patient outcomes. With the continuous evolution of imaging and robotic systems, their integration into neurosurgical procedures, particularly for brain tumor resection, has proven to be a pivotal development. For instance, the NeuroBlate system, which integrates intraoperative MRI with robotics, has been employed in over 3,500 successful procedures, demonstrating the critical role of real-time imaging in enhancing surgical accuracy. This integration has addressed previous challenges, such as the lack of intraoperative imaging, by improving material selection, sensor integration, and control mechanisms, thereby optimizing surgical results [182].

The effectiveness of robotic systems in brain tumor resection is closely linked to the surgeon's experience and familiarity with these technologies. A comprehensive review of 3,901 patients revealed significant variations in the learning curve, with 65% of studies noting a reduction in operative time as surgeons became more proficient with robotic systems. This reduction is crucial as it correlates with shorter anesthesia duration and potentially fewer complications, emphasizing the importance of mastering robotic technologies for improved surgical outcomes. However, the variability in learning curves across different studies indicates that the benefits of robotic systems are not universally guaranteed and depend heavily on the complexity of the procedure and the surgeon's expertise [183].

Despite the clear advantages, the adoption of robotic systems in neurosurgery is not without challenges, particularly regarding ethical and legal considerations. The precision and capabilities of robotic technologies introduce new ethical dilemmas, especially in terms of patient autonomy and informed consent. Additionally, the evolving medicolegal landscape demands a redefinition of the standard of care as robotic technologies advance. The MAUDE database recorded 144 deaths and 1,391 patient injuries related to robotic systems between 2000 and 2013, highlighting the need for rigorous monitoring and reporting to ensure patient safety [184]. Therefore, while robotic technologies hold great promise, their implementation in brain tumor resection must be accompanied by careful ethical and legal considerations.

In the context of skull base surgery, robotic technologies, particularly the da Vinci Surgical System, have shown potential in procedures like transoral robotic surgery (TORS). These systems offer enhanced 3D visualization and precise manipulation, which are crucial in navigating complex anatomical regions such as the sella turcica. However, the current size and design of robotic instruments, initially developed for other surgical specialties, pose challenges in delicate skull base procedures, underscoring the need for future innovations focused on miniaturization and specialized tools [185]. Despite these limitations, the potential for improved outcomes in brain tumor resection remains significant as these technologies continue to evolve.

The adoption of robotic technologies for brain tumor resection in low- and middle-income countries (LMICs) is constrained by financial and infrastructural challenges. The scarcity of neurosurgeons, limited access to training, and insufficient funding for research further hinder the widespread use of these advanced technologies. Nevertheless, promoting the use of robotic systems in these regions is vital for improving neurosurgical outcomes and advancing global health equity. The COVID-19 pandemic has underscored the necessity of robust neurosurgical capabilities, especially in managing post-COVID-19 neurological syndromes, which are not well understood and present additional challenges in LMICs [186]. Therefore, international collaboration and investment in neurosurgical infrastructure are essential to support the broader adoption of robotic technologies in these regions. Finally, the economic impact of using robotic technologies in brain tumor resection remains a topic of ongoing debate. While these technologies have been shown to improve surgical ergonomics and precision, leading to better patient outcomes, their cost-effectiveness is still under scrutiny. Some studies suggest that reducing operative time by 20-30 minutes could save approximately $1,860-$2,790 per surgery, highlighting the financial benefits of increased efficiency [187]. However, further research is needed to fully understand the economic implications of using robotic systems in complex neurosurgical procedures. The future of robotic technologies in brain tumor resection is promising, with emerging innovations such as haptic feedback, tele-guided systems, and AI-driven tools expected to further enhance surgical precision and safety. As these technologies continue to advance, they are likely to become integral to neurosurgical practice, offering new possibilities for improving patient care and surgical outcomes [188, 189].

AI and Its Role in Neurosurgery

Artificial intelligence (AI) is the branch of computer science attempting to equip machines with human-like intelligence, enabling their ability to learn, reason, and problem-solve when presented with numerous different forms of data. Complex and intricate neurosurgical procedures make the field of brain tumor surgery an ideal candidate for greater integration of AI. Artificial intelligence can assist the patient in all stages of their surgical procedure, given that brain tumors are mostly asymptomatic. AI has been developed to early detect patients with this risk by detecting tumor markers in the blood, detecting the tumor even before MRI (The gold standard of brain tumor) in addition to intraoperative tissue analysis and intraoperative workflow analysis and staging the risk of the patient with central nervous system (CNS) tumors to perform surgery. Nevertheless, several barriers to widespread introduction exist such as a requirement of large datasets to train existing ML programs, selection bias of training data, and slow progress in advancing ML programming [190, 191].

The integration of augmented reality, virtual reality, robotic technologies, and artificial intelligence is fundamentally reshaping the landscape of neurosurgery, particularly in the resection of brain tumors. These advanced tools are not only enhancing preoperative planning and intraoperative precision but are also redefining expectations for postoperative outcomes.

In recent years, AR and VR have demonstrated extraordinary potential, not just in supporting surgeons during procedures but also in revolutionizing preoperative training and preparation. The ability to immerse oneself in detailed simulations allows surgeons to gain a deeper understanding of individual anatomical complexities, thereby reducing the risks associated with highly delicate interventions. Simultaneously, the introduction of robotic systems has marked a pivotal shift, leading to greater precision in tumor resections. Operations that were once limited by human hands now benefit from millimeter-level control, minimizing damage to surrounding healthy tissue and accelerating postoperative recovery. However, despite the evident technical superiority, integrating these machines into operating rooms presents a series of ethical and practical challenges that require ongoing reflection within the medical community. Looking ahead, the potential of artificial intelligence cannot be overlooked, as it is rapidly becoming a crucial element at every stage of the surgical process. AI is no longer merely a diagnostic aid; it is quickly becoming an indispensable tool in the planning and personalization of treatment, refining surgical strategies in real-time and tailoring them to the specific needs of each patient. Future prospects suggest an increasing integration of these technologies, which will not only continue to improve the precision and safety of surgical procedures but also potentially reduce operating time and overall costs. However, the true potential lies in their combination: the synergy between AR, VR, robotics, and AI promises to open new frontiers in neurosurgery, offering possibilities that, until recently, were considered unattainable. Ultimately, the future of neurosurgery will not be defined by a single innovation but by the convergence of these cutting-edge technologies, which together will revolutionize the way brain tumors are treated, with a lasting impact on patients' lives and the medical field as a whole.

## Conclusions

The advancements in neurosurgical techniques for brain tumor resection have significantly improved patient outcomes in terms of neurological function and survival rates. From traditional approaches like craniotomy to minimally invasive procedures such as endoscopic endonasal surgeries and stereotactic radiosurgery, the field of neurosurgery has made remarkable progress.

Despite the challenges, the integration of advanced imaging techniques, the development of refined surgical tools, and the potential application of artificial intelligence in surgical planning are expected to further enhance the safety and efficacy of neurosurgical procedures. It is important to note that continued research and innovation in this field, as highlighted in this comprehensive review, hold great promise for improving the management of brain tumors and ultimately benefiting patients.

## References

[1] Miller KD, Ostrom QT, Kruchko C (2021). Brain and other central nervous system tumor statistics, 2021. CA Cancer J Clin.

[2] Nature Reviews Cancer (2020). Focusing on brain tumours and brain metastasis. Nat Rev Cancer.

[3] Ostrom QT, Patil N, Cioffi G, Waite K, Kruchko C, Barnholtz-Sloan JS (2020). CBTRUS statistical report: primary brain and other central nervous system tumors diagnosed in the United States in 2013-2017. Neuro Oncol.

[4] Zada G, Bond AE, Wang YP, Giannotta SL, Deapen D (2012). Incidence trends in the anatomic location of primary malignant brain tumors in the United States: 1992-2006. World Neurosurg.

[5] Tan AC, Ashley DM, López GY, Malinzak M, Friedman HS, Khasraw M (2020). Management of glioblastoma: state of the art and future directions. CA Cancer J Clin.

[6] Hatoum R, Chen JS, Lavergne P (2022). Extent of tumor resection and survival in pediatric patients with high-grade gliomas: a systematic review and meta-analysis. JAMA Netw Open.

[7] Khan S, Luqman S (2023). Neurosurgical Techniques for Brain Tumor Removal: Minimally Invasive Approaches and Functional Mapping. https://www.researchgate.net/publication/372410186_Neurosurgical_Techniques_for_Brain_Tumor_Removal_Minimally_Invasive_Approaches_and_Functional_Mapping.

[8] Feigl GC, Staribacher D, Britz G, Kuzmin D (2024). Minimally invasive approaches in the surgical treatment of intracranial meningiomas: an analysis of 54 cases. Brain Tumor Res Treat.

[9] Tejada Solís S, de Quintana Schmidt C, Gonzalez Sánchez J, Fernández Portales I, Del Álamo de Pedro M, Rodríguez Berrocal V, Díez Valle R (2020). Intraoperative imaging in the neurosurgery operating theatre: a review of the most commonly used techniques for brain tumour surgery. Neurocirugia.

[10] Park J, Park YG (2022). Brain tumor rehabilitation: symptoms, complications, and treatment strategy. Brain Neurorehabil.

[11] Duffau H (2013). A new philosophy in surgery for diffuse low-grade glioma (DLGG): oncological and functional outcomes. Neurochirurgie.

[12] Spina A, Boari N, Gagliardi F, Bailo M, Iannaccone S, Mortini P (2019). Gamma Knife radiosurgery for trigeminal neuralgia: when?. Neurosurg Rev.

[13] Cappabianca P, Cavallo LM, Solari D, Stagno V, Esposito F, de Angelis M (2014). Endoscopic endonasal surgery for pituitary adenomas. World Neurosurg.

[14] Takeda J, Nonaka M, Li Y, Isozaki H, Kamei T, Hashiba T, Asai A (2022). 5-Aminolevulinic acid fluorescence-guided endoscopic surgery for intraventricular tumors. Surg Neurol Int.

[15] Hervey-Jumper SL, Li J, Lau D, Molinaro AM, Perry DW, Meng L, Berger MS (2015). Awake craniotomy to maximize glioma resection: methods and technical nuances over a 27-year period. J Neurosurg.

[16] Shiue K, Sahgal A, Lo SS (2023). Precision radiation for brain metastases with a focus on hypofractionated stereotactic radiosurgery. Semin Radiat Oncol.

[17] Hawasli AH, Bagade S, Shimony JS, Miller-Thomas M, Leuthardt EC (2013). Magnetic resonance imaging-guided focused laser interstitial thermal therapy for intracranial lesions: single-institution series. Neurosurgery.

[18] Ratre S, Yadav YR, Parihar VS, Kher Y (2016). Microendoscopic removal of deep-seated brain tumors using tubular retraction system. J Neurol Surg A Cent Eur Neurosurg.

[19] Pichardo-Rojas PS, Angulo-Lozano JC, Alvarez-Castro JA (2024). Intraoperative magnetic resonance imaging (MRI)-guided resection of glioblastoma: a meta-analysis of 1,847 patients. World Neurosurg.

[20] Altawalbeh G, Goldberg M, Mondragón-Soto MG (2024). Navigating brain metastases: unveiling the potential of 3-tesla intraoperative magnetic resonance imaging. Cancers (Basel).

[21] Mazzucchi E, Cavlak LB, Pignotti F (2024). Evaluation of the extent of resection of intracranial tumors with virtual intraoperative MRI: a case series. J Neurosurg.

[22] Herrera RR, Ledesma JL, Rojas HP, Sanz F, Herrera JM, Estramiana A, Bottan JS (2024). Intraoperative magnetic resonance imaging in brain glioma surgery using low-field system. Presentation of the first twenty-eight procedures. Med Res Arch.

[23] Tuleasca C, Leroy HA, Peciu-Florianu I (2021). Impact of combined use of intraoperative MRI and awake microsurgical resection on patients with gliomas: a systematic review and meta-analysis. Neurosurg Rev.

[24] Choudhri AF, Siddiqui A, Klimo P Jr, Boop FA (2015). Intraoperative MRI in pediatric brain tumors. Pediatr Radiol.

[25] Schichor C, Terpolilli N, Thorsteinsdottir J, Tonn JC (2017). Intraoperative computed tomography in cranial neurosurgery. Neurosurg Clin N Am.

[26] Noh T, Mustroph M, Golby AJ (2021). Intraoperative imaging for high-grade glioma surgery. Neurosurg Clin N Am.

[27] Riva M, Hiepe P, Frommert M (2020). Intraoperative computed tomography and finite element modelling for multimodal image fusion in brain surgery. Oper Neurosurg (Hagerstown).

[28] Jermakowicz WJ, Diaz RJ, Cass SH, Ivan ME, Komotar RJ (2016). Use of a mobile intraoperative computed tomography scanner for navigation registration during laser interstitial thermal therapy of brain tumors. World Neurosurg.

[29] Saß B, Pojskic M, Bopp M, Nimsky C, Carl B (2021). Comparing fiducial-based and intraoperative computed tomography-based registration for frameless stereotactic brain biopsy. Stereotact Funct Neurosurg.

[30] Fuad NA, Alias A, Rosli FJ, Idris Z, Ismail R, Anour AA (2024). A comparison of intraoperative ultrasound and post-operative MRI in paediatric intra axial tumours [PREPRINT]. Res Square.

[31] Del Bene M, DiMeco F, Unsgård G (2021). Editorial: Intraoperative ultrasound in brain tumor surgery: state-of-the-art and future perspectives. Front Oncol.

[32] Moiyadi AV, Shetty P, Degaonkar A (2017). Resection of pediatric brain tumors: intraoperative ultrasound revisited. J Pediatr Neurosci.

[33] Incekara F, Smits M, Dirven L (2021). Intraoperative B-mode ultrasound guided surgery and the extent of glioblastoma resection: a randomized controlled trial. Front Oncol.

[34] Klein Gunnewiek K, van Baarsen KM, Graus EH, Brink WM, Lequin MH, Hoving EW (2024). Navigated intraoperative ultrasound in pediatric brain tumors. Childs Nerv Syst.

[35] Elmesallamy WA (2024). Gross pathology of brain mass lesions by intraoperative ultrasonography: a comparative study. Egypt J Neurosurg.

[36] Prada F, Del Bene M, Rampini A (2019). Intraoperative strain elastosonography in brain tumor surgery. Oper Neurosurg (Hagerstown).

[37] Del Bene M, Perin A, Casali C (2018). Advanced ultrasound imaging in glioma surgery: beyond gray-scale B-mode. Front Oncol.

[38] Saß B, Pojskic M, Zivkovic D, Carl B, Nimsky C, Bopp MH (2021). Utilizing intraoperative navigated 3D color Doppler ultrasound in glioma surgery. Front Oncol.

[39] Saß B, Carl B, Pojskic M, Nimsky C, Bopp M (2020). Navigated 3D ultrasound in brain metastasis surgery: analyzing the differences in object appearances in ultrasound and magnetic resonance imaging. Appl Sci (Switzerland).

[40] Prada F, Ciocca R, Corradino N (2022). Multiparametric intraoperative ultrasound in oncological neurosurgery: a pictorial essay. Front Neurosci.

[41] Ajmal S (2021). Contrast-enhanced ultrasonography: review and applications. Cureus.

[42] Prada F, Del Bene M, Mauri G (2018). Dynamic assessment of venous anatomy and function in neurosurgery with real-time intraoperative multimodal ultrasound: technical note. Neurosurg Focus.

[43] Della Pepa GM, Ius T, La Rocca G (2020). 5-Aminolevulinic acid and contrast-enhanced ultrasound: the combination of the two techniques to optimize the extent of resection in glioblastoma surgery. Neurosurgery.

[44] Zhang Y, Sun X, Li J (2022). The diagnostic value of contrast-enhanced ultrasound and superb microvascular imaging in differentiating benign from malignant solid breast lesions: a systematic review and meta-analysis. Clin Hemorheol Microcirc.

[45] Cai S, Xing H, Wang Y (2024). Clinical application of intraoperative ultrasound superb microvascular imaging in brain tumors resections: contributing to the achievement of total tumoral resection. BMC Med Imaging.

[46] Cepeda S, García-García S, Arrese I (2021). Comparison of intraoperative ultrasound B-mode and strain elastography for the differentiation of glioblastomas from solitary brain metastases. An automated deep learning approach for image analysis. Front Oncol.

[47] Della Pepa GM, Menna G, Stifano V (2021). Predicting meningioma consistency and brain-meningioma interface with intraoperative strain ultrasound elastography: a novel application to guide surgical strategy. Neurosurg Focus.

[48] Menna G, Olivi A, Della Pepa GM (2021). Integration of different intraoperative ultrasound modalities in meningioma surgery: a 4-step approach. World Neurosurg.

[49] Šteňo A, Buvala J, Babková V, Kiss A, Toma D, Lysak A (2021). Current limitations of intraoperative ultrasound in brain tumor surgery. Front Oncol.

[50] Brahimaj BC, Kochanski RB, Pearce JJ (2021). Structural and functional imaging in glioma management. Neurosurgery.

[51] Chen JE, Glover GH (2015). Functional magnetic resonance imaging methods. Neuropsychol Rev.

[52] Al-Arfaj HK, Al-Sharydah AM, AlSuhaibani SS, Alaqeel S, Yousry T (2023). Task-based and resting-state functional MRI in observing eloquent cerebral areas personalized for epilepsy and surgical oncology patients: a review of the current evidence. J Pers Med.

[53] Lv H, Wang Z, Tong E (2018). Resting-state functional MRI: everything that nonexperts have always wanted to know. AJNR Am J Neuroradiol.

[54] Sahu A, Kurki V, Vijan A, Janu A, Shetty P, Moiyadi A (2021). Case series of applications of resting state functional MRI in brain tumor surgery: a novel technique. Indian J Radiol Imaging.

[55] Manan HA, Franz EA, Yahya N (2020). Functional connectivity changes in patients with brain tumours—A systematic review on resting state-fMRI. Neurol Psychiatry Brain Res.

[56] Liu X, Li J, Xu Q (2022). RP-Rs-fMRIomics as a novel imaging analysis strategy to empower diagnosis of brain gliomas. Cancers (Basel).

[57] Yahyavi-Firouz-Abadi N, Pillai JJ, Lindquist MA (2017). Presurgical brain mapping of the ventral somatomotor network in patients with brain tumors using resting-state fMRI. AJNR Am J Neuroradiol.

[58] Morrison MA, Churchill NW, Cusimano MD, Schweizer TA, Das S, Graham SJ (2016). Reliability of task-based fMRI for preoperative planning: a test-retest study in brain tumor patients and healthy controls. PLoS One.

[59] Stippich C, Blatow M, Garcia M (2015). Task-based presurgical functional MRI in patients with brain tumors. Clinical Functional MRI. Medical Radiology.

[60] Lakhani DA, Sabsevitz DS, Chaichana KL, Quiñones-Hinojosa A, Middlebrooks EH (2023). Current state of functional MRI in the presurgical planning of brain tumors. Radiol Imaging Cancer.

[61] Nandakumar N, Manzoor K, Agarwal S, Pillai JJ, Gujar SK, Sair HI, Venkataraman A (2021). Automated eloquent cortex localization in brain tumor patients using multi-task graph neural networks. Med Image Anal.

[62] Holzgreve A, Albert NL, Galldiks N, Suchorska B (2021). Use of PET imaging in neuro-oncological surgery. Cancers (Basel).

[63] Karlberg A, Berntsen EM, Johansen H (2019). 18F-FACBC PET/MRI in diagnostic assessment and neurosurgery of gliomas. Clin Nucl Med.

[64] Apra C, Bemora JS, Palfi S (2024). Achieving gross total resection in neurosurgery: a review of intraoperative techniques and their influence on surgical goals. World Neurosurg.

[65] Borja AJ, Hancin EC, Raynor WY (2021). A critical review of PET tracers used for brain tumor imaging. PET Clin.

[66] Brendle C, Maier C, Bender B (2022). Impact of (18)F-FET PET/MRI on clinical management of brain tumor patients. J Nucl Med.

[67] Gutsche R, Lowis C, Ziemons K (2023). Automated brain tumor detection and segmentation for treatment response assessment using amino acid PET. J Nucl Med.

[68] Kersch CN, Ambady P, Hamilton BE, Barajas RF Jr (2022). MRI and PET of brain tumor neuroinflammation in the era of immunotherapy, from the AJR special series on inflammation. AJR Am J Roentgenol.

[69] Li Z, Chen J, Kong Z (2024). Correction to: A bis-boron boramino acid PET tracer for brain tumor diagnosis. Eur J Nucl Med Mol Imaging.

[70] Overcast WB, Davis KM, Ho CY, Hutchins GD, Green MA, Graner BD, Veronesi MC (2021). Advanced imaging techniques for neuro-oncologic tumor diagnosis, with an emphasis on PET-MRI imaging of malignant brain tumors. Curr Oncol Rep.

[71] Yamaki T, Higuchi Y, Yokota H (2022). The role of optimal cut-off diagnosis in 11C-methionine PET for differentiation of intracranial brain tumor from non-neoplastic lesions before treatment. Clin Imaging.

[72] Pamir MN, Özduman K, Yıldız E, Sav A, Dinçer A (2013). Intraoperative magnetic resonance spectroscopy for identification of residual tumor during low-grade glioma surgery: clinical article. J Neurosurg.

[73] Roder C, Skardelly M, Ramina KF (2014). Spectroscopy imaging in intraoperative MR suite: tissue characterization and optimization of tumor resection. Int J Comput Assist Radiol Surg.

[74] Grech-Sollars M, Vaqas B, Thompson G, Barwick T, Honeyfield L, O'Neill K, Waldman AD (2017). An MRS- and PET-guided biopsy tool for intraoperative neuronavigational systems. J Neurosurg.

[75] McCarthy L, Verma G, Hangel G (2022). Application of 7T MRS to high-grade gliomas. AJNR Am J Neuroradiol.

[76] Wang L, Chen G, Dai K (2022). Hydrogen proton magnetic resonance spectroscopy (MRS) in differential diagnosis of intracranial tumors: a systematic review [RETRACTED]. Contrast Media Mol Imaging.

[77] Toh CH, Castillo M, Wei KC, Chen PY (2020). MRS as an aid to diagnose malignant transformation in low-grade gliomas with increasing contrast enhancement. AJNR Am J Neuroradiol.

[78] Hu X, Xue M, Sun S (2021). Combined application of MRS and DWI can effectively predict cell proliferation and assess the grade of glioma: a prospective study. J Clin Neurosci.

[79] Travers S, Joshi K, Miller DC (2021). Reliability of magnetic resonance spectroscopy and positron emission tomography computed tomography in differentiating metastatic brain tumor recurrence from radiation necrosis. World Neurosurg.

[80] Mulyadi R, Islam AA, Murtala B, Tammase J, Hatta M, Firdaus M (2020). Diagnostic yield of the combined magnetic resonance imaging and magnetic resonance spectroscopy to predict malignant brain tumor. Bali Med J.

[81] Picart T, Gautheron A, Caredda C, Ray C, Mahieu-Williame L, Montcel B, Guyotat J (2024). Fluorescence-guided surgical techniques in adult diffuse low-grade gliomas: state-of-the-art and emerging techniques: a systematic review. Cancers (Basel).

[82] Chicoine MR, Sylvester P, Yahanda AT, Shah A (2020). Image guidance in cranial neurosurgery: how a six-ton magnet and fluorescent colors make brain tumor surgery better. Mo Med.

[83] Batalov AI, Goryaynov SA, Zakharova NE, Solozhentseva KD, Kosyrkova AV, Potapov AA, Pronin IN (2021). Prediction of intraoperative fluorescence of brain gliomas: correlation between tumor blood flow and the fluorescence. J Clin Med.

[84] Orillac C, Stummer W, Orringer DA (2021). Fluorescence guidance and intraoperative adjuvants to maximize extent of resection. Neurosurgery.

[85] Alcazar P, Avedillo A, Vazquez S (2023). The usefulness of intraoperative sodium fluorescein in the surgical treatment of relapsed high-grade brain tumors in pediatric patients. Childs Nerv Syst.

[86] Di Cristofori A, Carone G, Rocca A, Rui CB, Trezza A, Carrabba G, Giussani C (2023). Fluorescence and intraoperative ultrasound as surgical adjuncts for brain metastases resection: what do we know? A systematic review of the literature. Cancers (Basel).

[87] Yandrapalli S, Puckett Y (2024). SPECT imaging. In: StatPearls [Internet].

[88] Alam SS, Junaid S, Ahmed SM (2016). Evaluation of Technetium-99m glucoheptonate single photon emission computed tomography for brain tumor grading. Asian J Neurosurg.

[89] Kumar P, Kumar A, Nagaraj C (2024). Evaluating the diagnostic efficacy of (99m)Tc-methionine single-photon emission computed tomography-computed tomography: a head-to-head comparison with (11)C-methionine positron emission tomography-magnetic resonance imaging in glioma patients. Cancer Biother Radiopharm.

[90] Khangembam BC, Singhal A, Kumar R, Bal C (2019). Tc-99m glucoheptonate single photon emission computed tomography-computed tomography for detection of recurrent glioma: a prospective comparison with N-13 ammonia positron emission tomography-computed tomography. Indian J Nucl Med.

[91] Bouchareb Y, AlSaadi A, Zabah J (2024). Technological advances in SPECT and SPECT/CT imaging. Diagnostics.

[92] Zhang J, Traylor KS, Mountz JM (2020). PET and SPECT imaging of brain tumors. Semin Ultrasound CT MR.

[93] Rigante L, Borghei-Razavi H, Recinos PF, Roser F (2019). An overview of endoscopy in neurologic surgery. Cleve Clin J Med.

[94] Kassam AB, Gardner P, Snyderman C, Mintz A, Carrau R (2005). Expanded endonasal approach: fully endoscopic, completely transnasal approach to the middle third of the clivus, petrous bone, middle cranial fossa, and infratemporal fossa. Neurosurg Focus.

[95] Ethiraj S, Varma V, Subhash S (2024). Endoscopic Neurosurgery in the 21st Century: A Comprehensive Review of Challenges and Prospects. https://www.sdiarticle5.com/review-history/112958.

[96] El Beltagy MA, Atteya MM (2021). Benefits of endoscope-assisted microsurgery in the management of pediatric brain tumors. Neurosurg Focus.

[97] Uvelius E, Siesjö P (2020). 3-D endoscopy in surgery of pituitary adenomas, prospective evaluation of patient gain using basic outcome parameters. J Clin Neurosci.

[98] Matsumoto Y, Kurozumi K, Shimazu Y, Ichikawa T, Date I (2016). Endoscope-assisted resection of cavernous angioma at the foramen of Monro: a case report. SpringerPlus.

[99] Alalade AF, Ogando-Rivas E, Boatey J, Souweidane MM, Anand VK, Greenfield JP, Schwartz TH (2018). Suprasellar and recurrent pediatric craniopharyngiomas: expanding indications for the extended endoscopic transsphenoidal approach. J Neurosurg Pediatr.

[100] Sankhla SK, Warade A, Khan GM (2024). Endoport-guided endoscopic excision of intraaxial brain tumors. In: Endoscope-controlled Transcranial Surgery. Advances and Technical Standards in Neurosurgery.

[101] Dziedzic T, Bernstein M (2014). Awake craniotomy for brain tumor: indications, technique and benefits. Expert Rev Neurother.

[102] Richardson AM, McCarthy DJ, Sandhu J (2019). Predictors of successful discharge of patients on postoperative day 1 after craniotomy for brain tumor. World Neurosurg.

[103] Ma R, Livermore LJ, Plaha P (2016). Fast track recovery program after endoscopic and awake intraparenchymal brain tumor surgery. World Neurosurg.

[104] Brown T, Shah AH, Bregy A (2013). Awake craniotomy for brain tumor resection: the rule rather than the exception?. J Neurosurg Anesthesiol.

[105] Gupta DK, Chandra PS, Ojha BK, Sharma BS, Mahapatra AK, Mehta VS (2007). Awake craniotomy versus surgery under general anesthesia for resection of intrinsic lesions of eloquent cortex--a prospective randomised study. Clin Neurol Neurosurg.

[106] Sacko O, Lauwers-Cances V, Brauge D, Sesay M, Brenner A, Roux FE (2011). Awake craniotomy vs surgery under general anesthesia for resection of supratentorial lesions. Neurosurgery.

[107] Sattari SA, Rincon-Torroella J, Sattari AR (2024). Awake versus asleep craniotomy for patients with eloquent glioma: a systematic review and meta-analysis. Neurosurgery.

[108] Chowdhury T, Gray K, Sharma M (2022). Brain cancer progression: a retrospective multicenter comparison of awake craniotomy versus general anesthesia in high-grade glioma resection. J Neurosurg Anesthesiol.

[109] Groshev A, Padalia D, Patel S (2017). Clinical outcomes from maximum-safe resection of primary and metastatic brain tumors using awake craniotomy. Clin Neurol Neurosurg.

[110] Shah Z, Bakhshi SK, Khalil M, Shafiq F, Enam SA, Shamim MS (2023). Intraoperative seizures during awake craniotomy for brain tumor resection. Cureus.

[111] Starowicz-Filip A, Prochwicz K, Myszka A (2022). Subjective experience, cognitive functioning and trauma level of patients undergoing awake craniotomy due to brain tumor - Preliminary study. Appl Neuropsychol Adult.

[112] Fontaine D, Almairac F (2017). Pain during awake craniotomy for brain tumor resection. Incidence, causes, consequences and management. Neurochirurgie.

[113] Tan CL, Jain S, Chan HM, Loh NW, Teo K (2023). Awake craniotomy for brain tumor resection: Patient experience and acceptance in an Asian population. Asia Pac J Clin Oncol.

[114] Akay A, Islekel S (2018). Awake craniotomy procedure: its effects on neurological morbidity and recommendations. Turk Neurosurg.

[115] Kurian J, Pernik MN, Traylor JI, Hicks WH, El Shami M, Abdullah KG (2022). Neurological outcomes following awake and asleep craniotomies with motor mapping for eloquent tumor resection. Clin Neurol Neurosurg.

[116] Fang S, Li Y, Wang Y, Zhang Z, Jiang T (2020). Awake craniotomy for gliomas involving motor-related areas: classification and function recovery. J Neurooncol.

[117] Bonifazi S, Passamonti C, Vecchioni S (2020). Cognitive and linguistic outcomes after awake craniotomy in patients with high-grade gliomas. Clin Neurol Neurosurg.

[118] Clavreul A, Aubin G, Delion M, Lemée JM, Ter Minassian A, Menei P (2021). What effects does awake craniotomy have on functional and survival outcomes for glioblastoma patients?. J Neurooncol.

[119] Chen T, Mirzadeh Z, Chapple K, Lambert M, Ponce FA (2017). Complication rates, lengths of stay, and readmission rates in "awake" and "asleep" deep brain simulation. J Neurosurg.

[120] Kwinta BM, Myszka AM, Bigaj MM, Krzyżewski RM, Starowicz-Filip A (2021). Intra- and postoperative adverse events in awake craniotomy for intrinsic supratentorial brain tumors. Neurol Sci.

[121] Gerritsen JKW, Zwarthoed RH, Kilgallon JL (2022). Effect of awake craniotomy in glioblastoma in eloquent areas (GLIOMAP): a propensity score-matched analysis of an international, multicentre, cohort study. Lancet Oncol.

[122] Kshettry VR, Kenning TJ, Evans JJ, Farrell CJ (2019). 9 principles of minimally invasive keyhole surgery. In: Endoscopic and Keyhole Cranial Base Surgery.

[123] Reisch R, Perneczky A, Filippi R (2003). Surgical technique of the supraorbital key-hole craniotomy. Surg Neurol.

[124] Iacoangeli M, Nocchi N, Nasi D (2016). Minimally invasive supraorbital key-hole approach for the treatment of anterior cranial fossa meningiomas. Neurol Med Chir (Tokyo).

[125] Banu MA, Mehta A, Ottenhausen M (2016). Endoscope-assisted endonasal versus supraorbital keyhole resection of olfactory groove meningiomas: comparison and combination of 2 minimally invasive approaches. J Neurosurg.

[126] Ormond DR, Hadjipanayis CG (2013). The supraorbital keyhole craniotomy through an eyebrow incision: its origins and evolution. Minim Invasive Surg.

[127] Ong V, Brown NJ, Pennington Z (2023). The pterional keyhole craniotomy approach: a historical perspective. World Neurosurg.

[128] Reisch R, Stadie A, Kockro R, Gawish I, Schwandt E, Hopf N (2009). The minimally invasive supraorbital subfrontal key-hole approach for surgical treatment of temporomesial lesions of the dominant hemisphere. Minim Invasive Neurosurg.

[129] Avery MB, Mallari RJ, Barkhoudarian G, Kelly DF (2022). Supraorbital and mini-pterional keyhole craniotomies for brain tumors: a clinical and anatomical comparison of indications and outcomes in 204 cases. J Neurosurg.

[130] Lin YJ, Chen KT, Lee CC (2018). Anterior skull base tumor resection by transciliary supraorbital keyhole craniotomy: a single institutional experience. World Neurosurg.

[131] Thakur JD, Mallari RJ, Corlin A (2022). Critical appraisal of minimally invasive keyhole surgery for intracranial meningioma in a large case series. PLoS One.

[132] Seaman SC, Ali MS, Marincovich A, Li L, Walsh JE, Greenlee JD (2022). Minimally invasive approaches to anterior skull base meningiomas. J Neurol Surg B Skull Base.

[133] Zhou L, Jing X, Wang C (2024). Clinical application of transcranial neuroendoscopy combined with supraorbital keyhole approach in minimally invasive surgery of the anterior skull base. Sci Rep.

[134] Shahid AH, Butler D, Dyess G (2024). Supraorbital keyhole approaches in the first 3 years of practice: outcomes and lessons learned. Patient series. J Neurosurg Case Lessons.

[135] Merenzon MA, Mendez Valdez MJ, Chandar J (2024). Minimally invasive keyhole approach for supramaximal frontal glioma resections: technical note. J Neurosurg.

[136] Bander ED, Pandey A, Yan J (2024). Olfactory groove meningiomas: supraorbital keyhole versus orbitofrontal, frontotemporal, or bifrontal approaches. J Neurosurg.

[137] Amirjamshidi A (2019). Key hole craniotomy: when, where, and how to apply?. Asian J Neurosurg.

[138] Gurses ME, Gökalp E, Gecici NN (2024). Minimally invasive resection of intracranial lesions using tubular retractors: a single surgeon series. Clin Neurol Neurosurg.

[139] Eichberg DG, Buttrick S, Brusko GD, Ivan M, Starke RM, Komotar RJ (2018). Use of tubular retractor for resection of deep-seated cerebral tumors and colloid cysts: single surgeon experience and review of the literature. World Neurosurg.

[140] Marenco-Hillembrand L, Prevatt C, Suarez-Meade P, Ruiz-Garcia H, Quinones-Hinojosa A, Chaichana KL (2020). Minimally invasive surgical outcomes for deep-seated brain lesions treated with different tubular retraction systems: a systematic review and meta-analysis. World Neurosurg.

[141] Okasha M, Ineson G, Pesic-Smith J, Surash S (2021). Transcortical approach to deep-seated intraventricular and intra-axial tumors using a tubular retractor system: a technical note and review of the literature. J Neurol Surg A Cent Eur Neurosurg.

[142] Taylor Z, Gupta A, Mehta NH (2024). Evaluating the impact of tubular retractors in glioma surgery: a systematic review and meta-analysis. Clin Neurol Neurosurg.

[143] Moraes CA, da Sila Neto JA, Guedes BW, Oliveira AM, de Oliveira Santos BF (2024). Syringe port system as a tubular retractor technique for brain lesions: case series and review of the literature. Arq Bras Neurocir.

[144] Valarezo-Chuchuca A, Morejón-Hasing L, Wong-Achi X, Egas M (2021). Minimally invasive surgery with tubular retractor system for deep-seated or intraventricular brain tumors: report of 13 cases and technique description. Interdiscip Neurosurg.

[145] de Macêdo Filho LJ, Diógenes AV, Barreto EG (2022). Endoscopic endonasal resection of the medial wall of the cavernous sinus and its impact on outcomes of pituitary surgery: a systematic review and meta-analysis. Brain Sci.

[146] Roa Montes de Oca JC, Gonçalves Estella JM, Nieto-Librero AB (2022). Olfactory groove meningiomas: comprehensive assessment between the different microsurgical transcranial approaches and the endoscopic endonasal approaches, systematic review and metanalysis on behalf of the EANS skull base section. Brain Spine.

[147] Mastantuoni C, Cavallo LM, Esposito F (2022). Midline skull base meningiomas: transcranial and endonasal perspectives. Cancers (Basel).

[148] Liu JK, Silva NA, Sevak IA, Eloy JA (2018). Transbasal versus endoscopic endonasal versus combined approaches for olfactory groove meningiomas: importance of approach selection. Neurosurg Focus.

[149] Li K, Zhang J, Wang XS, Ye X, Zhao YL (2020). A systematic review of effects and complications after transsphenoidal pituitary surgery: endoscopic versus microscopic approach. Minim Invasive Ther Allied Technol.

[150] Na MK, Jang B, Choi KS (2022). Craniopharyngioma resection by endoscopic endonasal approach versus transcranial approach: a systematic review and meta-analysis of comparative studies. Front Oncol.

[151] Figueredo LF, Martínez AL, Suarez-Meade P (2023). Current role of endoscopic endonasal approach for craniopharyngiomas: a 10-year systematic review and meta-analysis comparison with the open transcranial approach. Brain Sci.

[152] Solari D, d'Avella E, Agresta G (2023). Endoscopic endonasal approach for infradiaphragmatic craniopharyngiomas: a multicentric Italian study. J Neurosurg.

[153] Sindwani R, Sreenath SB, Recinos PF (2024). Endoscopic endonasal approach to intraconal orbital tumors: outcomes and lessons learned. Laryngoscope.

[154] Mo J, Hasegawa H, Shin M (2024). Endoscopic endonasal approach is superior to transcranial approach for small to medium tuberculum sellae meningiomas in terms of visual outcome and complications: a retrospective study in a single center. World Neurosurg.

[155] Fu TS, Yao CM, Ziai H (2021). Cost-effectiveness of endoscopic endonasal vs transcranial approaches for olfactory groove meningioma. Head Neck.

[156] Gilbo P, Zhang I, Knisely J (2017). Stereotactic radiosurgery of the brain: a review of common indications. Chin Clin Oncol.

[157] Yang I, Udawatta M, Prashant GN (2019). Stereotactic radiosurgery for neurosurgical patients: a historical review and current perspectives. World Neurosurg.

[158] Chen WC, Baal UH, Baal JD, Pai JS, Boreta L, Braunstein SE, Raleigh DR (2021). Efficacy and safety of stereotactic radiosurgery for brainstem metastases: a systematic review and meta-analysis. JAMA Oncol.

[159] Krist DT, Naik A, Thompson CM, Kwok SS, Janbahan M, Olivero WC, Hassaneen W (2022). Management of brain metastasis. Surgical resection versus stereotactic radiotherapy: a meta-analysis. Neurooncol Adv.

[160] Palmer JD, Klamer BG, Ballman KV (2022). Association of long-term outcomes with stereotactic radiosurgery vs whole-brain radiotherapy for resected brain metastasis: a secondary analysis of the N107C/CEC.3 (Alliance for Clinical Trials in Oncology/Canadian Cancer Trials Group) randomized clinical trial. JAMA Oncol.

[161] Brown PD, Ballman KV, Cerhan JH (2017). Postoperative stereotactic radiosurgery compared with whole brain radiotherapy for resected metastatic brain disease (NCCTG N107C/CEC·3): a multicentre, randomised, controlled, phase 3 trial. Lancet Oncol.

[162] Benkhaled S, Schiappacasse L, Awde A, Kinj R (2024). Stereotactic radiosurgery and stereotactic fractionated radiotherapy in the management of brain metastases. Cancers (Basel).

[163] Lamba N, Muskens IS, DiRisio AC (2017). Stereotactic radiosurgery versus whole-brain radiotherapy after intracranial metastasis resection: a systematic review and meta-analysis. Radiat Oncol.

[164] Redmond KJ, Mehta M (2015). Stereotactic radiosurgery for glioblastoma. Cureus.

[165] Patel KR, Burri SH, Boselli D (2017). Comparing pre-operative stereotactic radiosurgery (SRS) to post-operative whole brain radiation therapy (WBRT) for resectable brain metastases: a multi-institutional analysis. J Neurooncol.

[166] Redmond KJ, Gui C, Benedict S (2021). Tumor control probability of radiosurgery and fractionated stereotactic radiosurgery for brain metastases. Int J Radiat Oncol Biol Phys.

[167] Gruber I, Weidner K, Treutwein M, Koelbl O (2023). Stereotactic radiosurgery of brain metastases: a retrospective study. Radiat Oncol.

[168] Loo M, Clavier JB, Attal Khalifa J, Moyal E, Khalifa J (2021). Dose-response effect and dose-toxicity in stereotactic radiotherapy for brain metastases: a review. Cancers (Basel).

[169] Pichardo-Rojas PS, Vázquez-Alva D, Alvarez-Castro JA (2024). Comparative effectiveness of frame-based and mask-based Gamma Knife stereotactic radiosurgery in brain metastases: a 509 patient meta-analysis. J Neurooncol.

[170] Rizzetto F, Bernareggi A, Rantas S, Vanzulli A, Vertemati M (2020). Immersive virtual reality in surgery and medical education: diving into the future. Am J Surg.

[171] Delion M, Klinger E, Bernard F, Aubin G, Minassian AT, Menei P (2020). Immersing patients in a virtual reality environment for brain mapping during awake surgery: safety study. World Neurosurg.

[172] Katsevman GA, Greenleaf W, García-García R, Perea MV, Ladera V, Sherman JH, Rodríguez G (2021). Virtual reality during brain mapping for awake-patient brain tumor surgery: proposed tasks and domains to test. World Neurosurg.

[173] Siyar S, Azarnoush H, Rashidi S, Del Maestro RF (2020). Tremor assessment during virtual reality brain tumor resection. J Surg Educ.

[174] Mofatteh M, Mashayekhi MS, Arfaie S (2023). Augmented and virtual reality usage in awake craniotomy: a systematic review. Neurosurg Rev.

[175] Kin T, Nakatomi H, Shono N, Nomura S, Saito T, Oyama H, Saito N (2017). Neurosurgical virtual reality simulation for brain tumor using high-definition computer graphics: a review of the literature. Neurol Med Chir (Tokyo).

[176] Mishra R, Narayanan MD, Umana GE, Montemurro N, Chaurasia B, Deora H (2022). Virtual reality in neurosurgery: beyond neurosurgical planning. Int J Environ Res Public Health.

[177] Casanova M, Clavreul A, Soulard G (2021). Immersive virtual reality and ocular tracking for brain mapping during awake surgery: prospective evaluation study. J Med Internet Res.

[178] Ammirati M (2022). Augmented reality in brain tumor surgery using the microscope focal point as the virtual pointer. Acta Neurochir (Wien).

[179] Azarnoush H, Alzhrani G, Winkler-Schwartz A (2015). Neurosurgical virtual reality simulation metrics to assess psychomotor skills during brain tumor resection. Int J Comput Assist Radiol Surg.

[180] Manjila S, Rosa B, Price K, Manjila R, Mencattelli M, Dupont PE (2023). Corrigendum to "Robotic Instruments Inside the MRI Bore: Key Concepts and Evolving Paradigms in Imaging-Enhanced Cranial Neurosurgery" [World Neurosurgery 176 (2023) 127-139/20021]. World Neurosurg.

[181] Shlobin NA, Huang J, Wu C (2022). Learning curves in robotic neurosurgery: a systematic review. Neurosurg Rev.

[182] Vilanilam GC, Venkat EH (2022). Editorial. Ethical nuances and medicolegal vulnerabilities in robotic neurosurgery. Neurosurg Focus.

[183] Pangal DJ, Cote DJ, Ruzevick J (2022). Robotic and robot-assisted skull base neurosurgery: systematic review of current applications and future directions. Neurosurg Focus.

[184] Karasin B, Hardinge T, Eskuchen L, Watkinson J (2022). Care of the patient undergoing robotic-assisted brain biopsy with stereotactic navigation: an overview. AORN J.

[185] Ortega-Sierra MG, Cuello-Torres OA, Jiménez-Arteaga A, Pérez-Benitez LM, Bolaño-Romero MP (2022). Robotic neurosurgery and post-COVID-19 neurological syndrome: two colossal challenges for low- and middle-income countries. J Neurosurg Sci.

[186] Fiani B, Quadri SA, Farooqui M, Cathel A, Berman B, Noel J, Siddiqi J (2020). Impact of robot-assisted spine surgery on health care quality and neurosurgical economics: a systemic review. Neurosurg Rev.

[187] Ball T, González-Martínez J, Zemmar A (2021). Robotic applications in cranial neurosurgery: current and future. Oper Neurosurg (Hagerstown).

[188] Mattei TA, Rodriguez AH, Sambhara D, Mendel E (2014). Current state-of-the-art and future perspectives of robotic technology in neurosurgery. Neurosurg Rev.

[189] Durrani S, Onyedimma C, Jarrah R (2022). The virtual vision of neurosurgery: how augmented reality and virtual reality are transforming the neurosurgical operating room. World Neurosurg.

[190] Li G, Patel NA, Burdette EC, Pilitsis JG, Su H, Fischer GS (2021). A fully actuated robotic assistant for MRI-guided precision conformal ablation of brain tumors. IEEE ASME Trans Mechatron.

[191] Williams S, Layard Horsfall H, Funnell JP (2021). Artificial intelligence in brain tumour surgery-An emerging paradigm. Cancers (Basel).

